# Activatable smart contrast agents for photoacoustic imaging

**DOI:** 10.1002/smo2.70024

**Published:** 2025-10-30

**Authors:** Donghyeon Oh, Jihye Lee, Suyoung Park, Juyoung Yoon, Won Jong Kim, Chulhong Kim

**Affiliations:** ^1^ Departments of Electrical Engineering Convergence IT Engineering, Mechanical Engineering, Medical Science and Engineering Pohang University of Science and Technology (POSTECH) Pohang Republic of Korea; ^2^ Department of Chemistry POSTECH Pohang Republic of Korea; ^3^ POSTECH‐CATHOLIC Biomedical Engineering Institute Seoul Republic of Korea; ^4^ Department of Chemistry and Nanoscience Ewha Womans University Seoul Republic of Korea; ^5^ Graduate Program in Innovative Biomaterials Convergence Ewha Womans University Seoul Republic of Korea

**Keywords:** activatable, photoacoustic contrast agent, photoacoustic imaging, probing, trigger

## Abstract

Photoacoustic imaging (PAI) uniquely merges the molecular contrast of optical absorption with the centimeter‐scale penetration afforded by ultrasound detection, enabling noninvasive visualization of deep physiology beyond the reach of purely optical modalities. Conventional photoacoustic (PA) contrast agents have been used to improve detection sensitivity relative to endogenous absorbers, but their fixed optical properties limit adaptation to heterogeneous or evolving disease microenvironments. Activatable PAI contrast agents overcome this drawback by switching their absorbance spectra in real time in response to exogenous triggers (near‐infrared light, heat, ultrasound, and electromagnetic fields) or endogenous microenvironmental cues (hypoxia, pH, reactive oxygen species, glutathione, and disease‐related enzymes). These dynamic features extend PAI beyond static signal enhancement, enabling user‐controlled activation, suppression of background interference, and access to functional or molecular biomarkers that conventional agents cannot provide. This review surveys recent advances in the molecular design, activation chemistry, and biomedical applications of these “activatable” probes, highlighting strategies for noninvasive diagnosis, multimodal imaging, remote actuation, and synergistic theranostics. Looking ahead, the next frontier lies in pathology‐tailored probes that can noninvasively reveal biomarkers inaccessible to current methods, as illustrated by early successes in atherosclerosis imaging. Achieving this translation will require optimizing probe safety and clearance, standardizing characterization protocols, and integrating hybrid imaging platforms with quantitative PA tomography to generate reliable diagnostic metrics.

## Graphical abstract

This review highlights recent advances in smart activatable photoacoustic imaging (PAI) contrast agents, which dynamically modulate their optical properties in response to external stimuli or microenvironmental cues. It discusses their molecular design, activation mechanisms, biomedical applications, and future prospects for clinically tailored use that leverages the unique strengths of PAI.

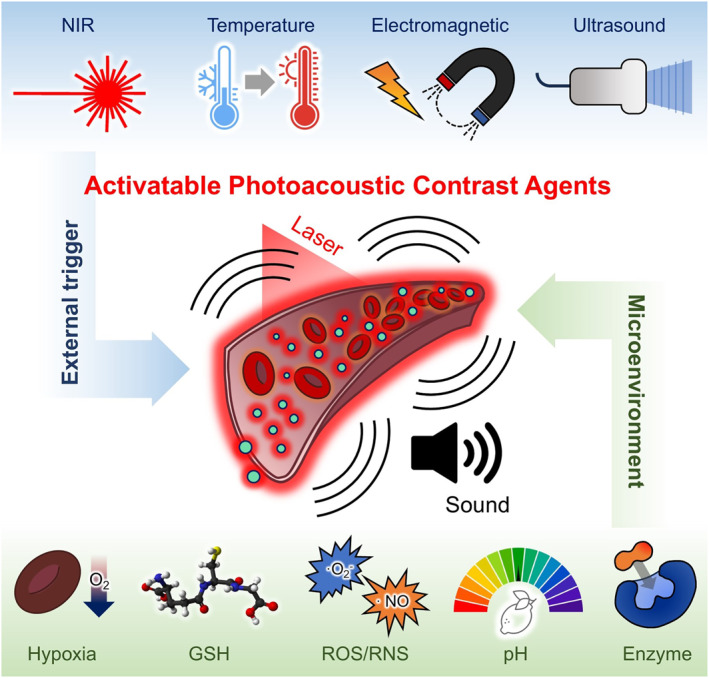

## 1 INTRODUCTION

Optical imaging techniques have revolutionized biomedical research by enabling visualization of cellular and molecular physiology in living tissues.^[1]^ Modalities such as confocal microscopy,^[2]^ multiphoton microscopy,^[3]^ and optical coherence tomography^[4]^ provide detailed structural and functional insights with subcellular precision. However, the inherent scattering and absorption of light in biological tissues severely limit the optical imaging depth, typically confining it to an optical ballistic regime of approximately 1–2 mm in the near‐infrared (NIR) window.[^5^, ^6^, ^7^] This fundamental limitation has spurred the development of hybrid imaging modalities that retain the molecular specificity of optical techniques while enabling deeper tissue penetration.[^8^, ^9^, ^10^] Among these, photoacoustic imaging (PAI) has emerged as a particularly promising approach that uniquely combines the rich contrast of optical absorption with the deep tissue penetration capabilities of ultrasound detection.[^11^, ^12^, ^13^, ^14^] PAI leverages the photoacoustic (PA) effect, in which the pulsed light absorbed by tissue chromophores induces rapid thermoelastic expansion, generating broadband acoustic waves.[^15^, ^16^, ^17^, ^18^] This combination of optical contrast and acoustic resolution makes PAI a powerful tool for noninvasive biomedical imaging, supporting a broad range of preclinical and clinical applications, including cancer detection,[^19^, ^20^, ^21^, ^22^] neurovascular imaging,[^23^, ^24^, ^25^, ^26^] and pathological vascular alterations[^27^, ^28^, ^29^, ^30^, ^31^, ^32^, ^33^].

The strength of PAI lies in its ability to generate cross‐sectional maps of endogenous optical absorbers such as melanin, hemoglobin, lipids and collagen across a broad optical spectrum.[^34^, ^35^, ^36^, ^37^, ^38^, ^39^, ^40^, ^41^, ^42^, ^43^] However, these endogenous signals often diminish with depth, limiting the effective imaging range of PAI to relatively shallow regions.[^44^, ^45^, ^46^, ^47^, ^48^, ^49^, ^50^, ^51^, ^52^] To overcome this challenge, contrast‐enhanced PAI with exogenous PA contrast agents has been developed to improve the imaging contrast and extend the depth.[^53^, ^54^, ^55^, ^56^, ^57^] Effective PA contrast agents must meet several essential criteria, such as strong optical absorption, high photothermal conversion efficiency, favorable biodistribution and clearance profiles, excellent biocompatibility, and robust photostability.[^58^, ^59^, ^60^, ^61^] More recently, research has shifted toward the functionalization of these agents for multimodal imaging, targeted delivery, and synergistic therapy, thereby broadening their clinical potential for diagnostic and therapeutic application^62^, ^63^, ^64^, ^65^, ^66^, ^67^, ^68^, ^69^.

**FIGURE 1:**
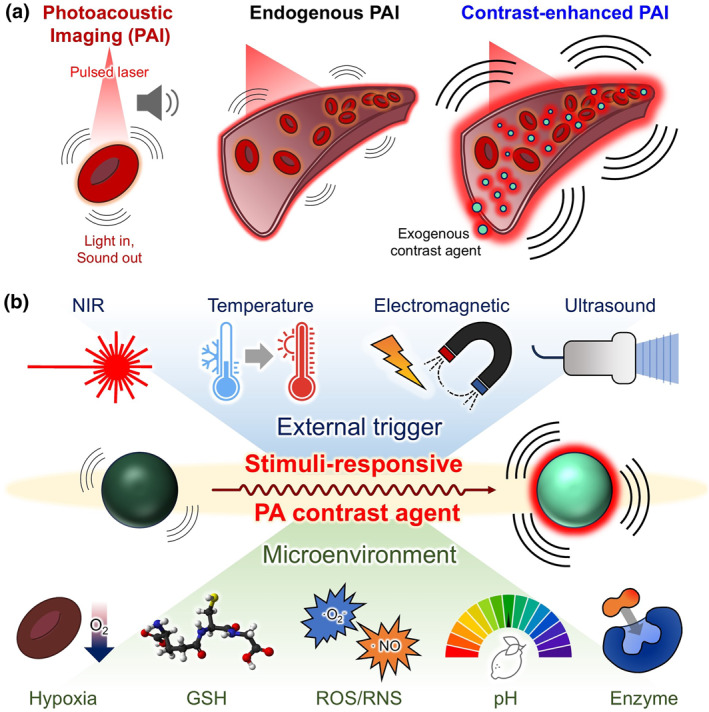
Contrast‐enhanced PAI using stimuli‐responsive PA contrast agents. (a) Schematic illustration of the photoacoustic effect, where pulsed laser irradiation generates acoustic signals, and of the enhancement achieved with PA contrast agents with high photothermal efficiency relative to endogenous chromophores. (b) Classification of stimuli‐responsive PA contrast agents based on their activation by either external triggers or microenvironmental biomarkers. PA, photoacoustic; PAI, photoacoustic imaging.

Building on this, smart or “responsive” PA contrast agents that dynamically alter their optical properties in response to specific external triggers or biological stimuli have attracted increasing interest (Figure 1). These agents can significantly enhance imaging specificity and functionality by providing real‐time feedback on environmental or pathological conditions. This review highlights recent advances in trigger‐responsive PA contrast agents that react to external stimuli and biological microenvironmental factors. We discuss the molecular design strategies and activation mechanisms of these agents and evaluate their potential to overcome the limitations of conventional PA probes while enhancing the precision and versatility of PAI.

## 2 CONVENTIONAL CONTRAST AGENTS

Based on their core compositions, the existing PA contrast agents can be classified into four categories: metallic nanoparticles, semiconducting nanoparticles, carbon nanomaterials, and organic dyes.[^70^, ^71^, ^72^, ^73^] The following sections provide detailed overviews of each of these categories.

### 2.1 Metallic nanoparticles

**FIGURE 2:**
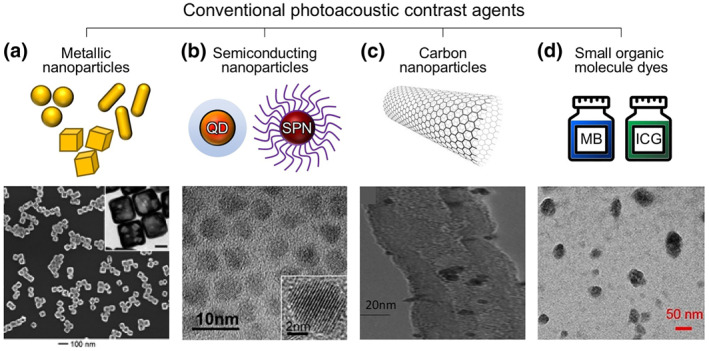
Categories of conventional photoacoustic contrast agents. (a) Metallic nanoparticles: Optically tunable 50 nm gold nanocages reveal the cerebral vasculature of small animals in vivo. Reproduced from ref. ^[74]^ with permission from American Chemical Society, copyright 2007. (b) Semiconducting nanoparticles: CuInS/ZnS quantum dots serve as theranostic nanomedicines for precise tumor localization and synergistic phototherapy. Reproduced from ref. ^[75]^ with permission from American Chemical Society, copyright 2016. (c) Carbon nanoparticles: Multiwalled carbon nanotubes anchored with RGD peptide–silica‐coated gold nanorods enable targeted photoacoustic imaging of in vivo gastric cancer cells. Reproduced from ref. ^[76]^ with permission from Springer Nature, copyright 2014. (d) Small organic molecule dyes: A nanostructured phthalocyanine complex (PcS4‐PcN4) formed via supramolecular self‐assembly exhibits enhanced PAI and photothermal therapy effect. Reproduced from ref. ^[77]^ with permission from Johns Wiley and Sons, copyright 2020. PA, photoacoustic; PAI, photoacoustic imaging; RGD, Arg‐Gly‐Asp.

Metallic nanoparticles have emerged as prominent PA contrast agents because of their exceptional optical properties, which are primarily attributed to the localized surface plasmon resonance (SPR) effect (Figure 2a).[^78^, ^79^] This phenomenon significantly enhances the optical absorption of these nanoparticles in the red and NIR spectral ranges, making them ideally suited for delivery through the depth of biological tissues. Among the various morphologies of metallic nanoparticles, gold‐based structures such as gold nanospheres, nanorods, nanoshells, and nanocages have gained particular attention because of their strong and tunable photothermal properties.^[80]^ Their photophysical properties depend strongly on their shape and size, attracting intense research attention to the tailored engineering of these form factors.^[81]^ Yang et al. introduced gold nanocages as novel contrast agents for PA tomography and used them to demonstrate enhanced imaging of the rat cerebral cortex vasculature, highlighting their potential for deep‐tissue biomedical imaging because of their tunable optical properties and large absorption cross‐section.^[74]^ Zhang et al. demonstrated the potential of gold nanoparticles as contrast agents for PA cancer imaging by using them to enhance tumor visualization in vivo and ex vivo in a mouse model of breast cancer.^[82]^ Song et al. pioneered the use of NIR gold nanocages for noninvasive PAI of sentinel lymph nodes, achieving a depth of ∼33 mm in a rat model with potential applications in breast cancer staging.^[83]^.

### 2.2 Semiconducting nanoparticles

Semiconducting nanoparticles, including inorganic quantum dots and polymeric semiconductor nanoparticles, exhibit optical properties and high photothermal conversion efficiency with superior robustness to photobleaching compared to that of traditional organic dyes, making them ideal for long‐term PAI applications (Figure 2b). Inorganic quantum dots, including iron oxide, copper sulfide, and manganese oxide nanoparticles, exploit the quantum confinement effect, in which decreasing particle size leads to more discrete energy levels and enhanced absorption properties.^[75]^ Shashkov et al. demonstrated that traditional fluorophore quantum dots (15–20 nm in diameter) function as effective PA and photothermal contrast agents and sensitizers with peak PA absorption at 640 nm, and reported that the polymer coating of quantum dots enhanced the PA intensity.^[84]^ The π‐electron delocalized backbones of semiconducting polymer nanoparticles (SPNs) enable strong NIR absorption and efficient photothermal conversion. Pu et al. developed ∼40 nm SPNs with superior photostability and higher PA signal output compared to those of carbon nanotubes and gold nanorods (AuNRs).^[85]^ They then successfully applied these SPNs in PAI for whole‐body lymph node mapping and reactive oxygen species (ROS) detection in live mice.

### 2.3 Carbon nanomaterials

Carbon nanomaterials in multiple allotropic forms, including graphene, carbon nanotubes, carbon dots, and other diverse types of carbon nanoparticles, have emerged as versatile and efficient PA contrast agents owing to their unique optical and physical properties^[76]^ (Figure 2c). The exceptional characteristics of carbon nanomaterials stem from their high optical absorption coefficients, excellent electrical and thermal conductivities, and efficient heat conversion capability. Furthermore, carbon nanomaterials exhibit strong reverse‐saturable absorption and self‐defocusing effects, which can be attributed to the presence of delocalized π‐electrons. The versatility of carbon nanomaterials is further enhanced by their ability to form diverse chemical bonds and undergo surface modifications, which allows their functionalization with drugs, inorganic materials, or organic compounds, thereby expanding their potential applications in theranostics and image‐guided therapies. De La Zerda et al. demonstrated that single‐walled carbon nanotubes conjugated with cyclic Arg‐Gly‐Asp (RGD) peptides can serve as effective targeted contrast agents for the PAI of tumors in living mice, showing eight times greater PA signals in tumors than the non‐targeted nanotubes.^[86]^ Pramanik et al. demonstrated a noninvasive method for sentinel lymph node identification using single‐walled carbon nanotube‐enhanced PAI in a rat model, achieving a high contrast‐to‐noise ratio of 89 at 793 nm excitation, revealing the potential of this method for future applications in molecular breast imaging.^[87]^

### 2.4 Small organic molecule dyes

Small organic molecules, particularly fluorophores and cyanine‐based photosensitizers, have emerged as crucial contrast agents for PAI (Figure 2d). This family includes porphyrins, cypates, phthalocyanines, naphthalocyanines, BODIPY dyes, and the IRDye700–800 series. These molecules exhibit narrow but exceptional absorption spectra in the NIR region, which enables deeper tissue penetration. With high molar extinction coefficients of up to 200,000 M^−1^ cm^−1^, they generate strong PA signals and offer versatility for structural modifications to enhance photostability, targeting ability, and therapeutic efficacy. Recent developments have focused on improving PA signal intensity through either self‐assembly or dye aggregation, increasing solubility via cargo delivery and surface engineering, and enhancing photostability with novel protective formulations.^[7]^ Notably, the clinical approval of indocyanine green (ICG) and methylene blue (MB) has accelerated the translation of contrast‐enhanced PAI due to their established safety profiles. Li et al. demonstrated a supramolecular self‐assembly approach using two water‐soluble phthalocyanine derivatives (PcS4 and PcN4), featuring enhanced PA signals and photothermal effects compared to those of the individual components, achieving successful in vivo tumor visualization and revealing the potential of this method for cancer photothermal therapy.^[77]^ Yang et al. developed a self‐assembled nanophthalocyanine (ZnPcN4‐TF) by combining a zinc(II) phthalocyanine derivative and transferrin for PAI‐guided phototherapy, demonstrating enhanced type I ROS production and photothermal efficiency under hypoxic conditions, with improved tumor accumulation and biocompatibility for effective cancer treatment.^[88]^.

## 3 STIMULI‐RESPONSIVE CONTRAST AGENTS

As discussed in the previous section, although conventional PA contrast agents possess fixed photochemical properties that are superior to those of endogenous chromophores, a new class of agents has emerged that enables dynamic contrast generation in response to changing biological environments.[^89^, ^90^, ^91^, ^92^, ^93^] These stimuli‐responsive agents can modulate their spectral properties in real time, offering enhanced functional and molecular specificity for PAI. Depending on the origin of the activating signal, they can be broadly divided into externally triggered agents, which allow user‐controlled, non‐invasive on/off modulation,^[94]^ and intrinsically triggered sensors, which respond to in vivo microenvironmental factors and function as molecular probes that reveal biochemical signatures within living systems, akin to activatable fluorescent dyes used in histological assays.^[95]^ In this section, we review recent advances in each category, organized by the type of stimulus used to activate PA contrast.

### 3.1 Externally triggered photoacoustic contrast agents

Although conventional PA contrast agents are limited by background signals from endogenous chromophores in biological tissues, externally triggered agents enable on‐demand activation, enabling more precise and temporally controlled imaging. In the following section, these agents are categorized based on the type of external stimulus—near‐infrared (NIR) light, temperature, ultrasound, and electromagnetic fields—and their activation mechanisms and biomedical applications are discussed with emphasis on enhanced imaging specificity and potential therapeutic synergy.

#### 3.1.1 Near‐infrared irradiation stimuli

**FIGURE 3:**
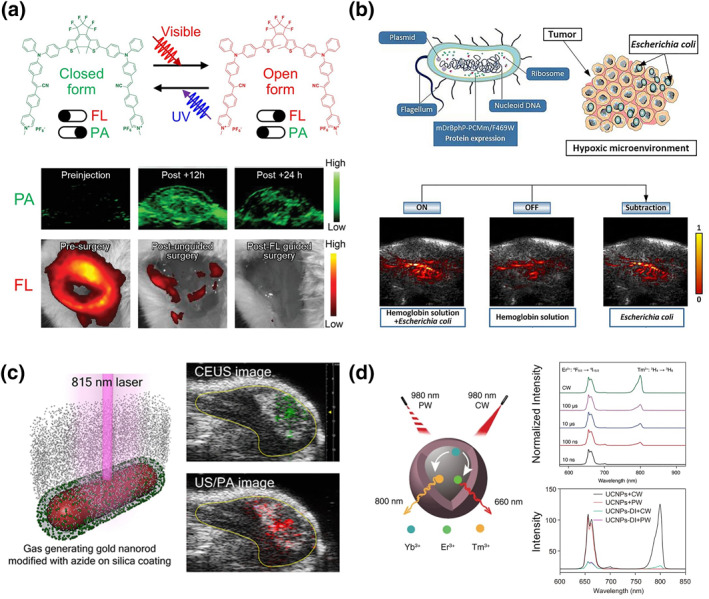
Near‐infrared (NIR) irradiation–responsive contrast agents. (a) Photoswitchable photosensitizer (DTE‐DPA‐SCP), which alternates between fluorescence (FL) mode (open‐ring form; 660 nm excitation) and photoacoustic (PA) mode (closed‐ring form; UV excitation). Both modalities were evaluated in the surgical removal of 4T1 tumors guided by in vivo PA/FL imaging. Reproduced from ref. ^[96]^ with permission from John Wiley & Sons, copyright 2023. (b) Genetically engineered phytochromic bacteria used as a reversibly switchable PA contrast agent (mDrBphP‐PCMm/F469W). Subtraction of sequential images acquired in the ON state (630 nm) and OFF state (780 nm) yields effective background PA suppression. Mapping of the hypoxic environment adjacent to the tumor was enabled by harnessing the anaerobic nature of the bacteria. Reproduced from ref. ^[97]^ with permission from National Academy of Sciences, copyright 2022. (c) Gas‐generating, laser‐activatable nanorods for contrast enhancement (GLANCE). PA‐induced photolysis of the azide‐containing capsule generates nitrogen bubbles that appear in both PA and contrast‐enhanced ultrasound (CEUS) images. Reproduced from ref. ^[98]^ with permission from American Chemical Society, copyright 2024. (d) Upconversion nanoparticles (UCNPs) for orthogonal separation of PA signal generation and phototherapy (continuous‐wave mode). Under 980 nm excitation, two distinct upconversion pathways are available: 660 nm red upconversion (Yb^3+^–Er^3+^) yielding pulsed waves (PW), and 800 nm NIR upconversion (Yb^3+^–Tm^3+^) yielding continuous waves (CW), thereby separating PA signal generation from reactive oxygen species (ROS) production. Reproduced from ref. ^[99]^ with permission from Springer Nature, copyright 2022. PA, photoacoustic.

Photodynamic therapy (PDT) is a comprehensive anticancer treatment that combines direct oxidative stress from photo‐induced ROS with immune responses triggered by immunogenic cell death. The administered photosensitizers typically exhibit strong optical absorption and may exhibit partial fluorescence (FL) or PA properties. However, conventional PDT has limitations, including potential damage to normal tissues, and suboptimal imaging contrast in the PA and FL modalities, owing to competitive energy pathways. Gao et al. developed a smart photosensitizer with ROS production and photophysical energy dissipation controlled by external NIR stimuli (Figure 3a).^[96]^ They synthesized DTE‐DPA‐SCP, where dithienylethene (DTE) is a photoswitchable ring unit that alternates between the ring‐opened form (RO), triggered by 660 nm light to provide FL, and the ring‐closed form (RC), triggered by UV light to provide photoacoustic contrast. The PA absorption spectra of the RO and RC forms differ significantly, with RC showing PA intensity proportional to concentration, whereas RO exhibited minimal signals. The potential of these NPs as function‐switchable nanoagents was tested using an in vivo PA/FL imaging‐guided tumor surgery model. 4T1 tumor‐bearing mice were injected with RC NPs, leading to increased PA contrast in tumors up to 12 h post‐injection, followed by a gradual decrease over 24 h. Tumors were then irradiated with a 660 nm laser to convert RC NPs into RO NPs, enabling intraoperative FL imaging for surgical tumor removal. Strong tumor‐specific FL contrast improved the precision of margin clearance compared with bright‐field imaging. Therapeutically, RO NPs combined with white light irradiation inhibited tumor growth. Flow cytometry revealed increased CD8^+^ T cells and mature dendritic cells, suggesting that the immunogenic cell death effect of PDT immunologically modulates the tumor microenvironment.

Photochromic chromoproteins called phytochromes are photoswitchable molecular structures occurring naturally in living microorganisms. The reversibly switchable characteristics of phytochromes have been harnessed to enhance PAI, significantly improving the molecular specificity through subtractive imaging techniques. This innovative approach was first explored by Yao et al.,^[100]^ Li et al.,^[101]^ and Stiel et al.,^[102]^ and on these studies, Gao et al. advanced the field by developing a hypoxic tumor‐targeted delivery system that utilizes genetically‐engineered anaerobic bacteria (*Escherichia coli*) expressing photochromic chromoproteins (Figure 3b).^[97]^ The absorption spectra of the chromoproteins (mDrBphP‐PCMm/F469W) were reversibly switchable between the ON and OFF states using 780 nm (ON to OFF) and 630 nm (OFF to ON) light. By acquiring PA images at 780 nm for ON and OFF states, the photoconversion ratio between these states was measured, thus effectively suppressing the background PA signals from endogenous chromophores (e.g., melanin, hemoglobin and lipids). They performed in vivo photoacoustic computed tomography imaging of mouse tumors, and their 7‐day longitudinal images demonstrated gradual colonization of *E. coli* (F469W) within the tumor, also observing tumor degradation in response to streptomycin antibiotic treatment.

Sun et al. discovered a novel method for light‐triggered gas formulation other than photothermal perfluorocarbon (PFC) vaporization via the photocatalytic function of gold nanoparticles, namely by using gas‐generating laser‐activatable nanorods for contrast enhancement (GLANCE, Figure 3c).^[98]^ Given that AuNRs exhibit excellent PA properties at the SPR peak in the NIR range, they induce the photolysis of encapsulating azide compounds and generate nitrogen gas bubbles that appear in PAI and ultrasound imaging (USI) diketopyrrolopyrrole polymer. They also revealed the change in the absorption spectra of GLANCE upon the laser irradiation, where its SPR peak at 815 nm vanished owing to the photofragmentation of AuNRs. This has further allowed differential imaging in PA and US modes with GLANCE, enhancing the molecular specificity of infiltrating PA/US contrasts in mouse tumors in vivo. PA and US images of tumor models displayed highly correlated distributions of GLANCE but showed opposite changes in the signal intensity before and after 815 nm pulsed laser irradiation, with a decrease in the PA signals and an increase in the US signals.

Yang et al. addressed the challenge of decoupling PAI and phototherapy during real‐time imaging by developing Yb/Tm/Er co‐doped upconversion nanoparticles (UCNPs) to orthogonally separate PAI and phototherapy (Figure 3d).^[99]^ PA signals are exclusively generated under pulsed wave (PW) laser excitation, whereas phototherapy is activated under continuous wave (CW) laser excitation. The UCNP complex utilized Yb^3+^–Er^3+^ pairs for red upconversion (660 nm) and Yb^3+^–Tm^3+^ pairs for NIR upconversion (800 nm), which responded to the pulse width of the 980 nm excitation laser. The UCNPs‐DI nanoagent incorporated diketopyrrolopyrrole polymer (to generate PA signals under 660 nm excitation) and ICG (to produce ROS under 800 nm excitation). UCNPs‐DI exhibited broad absorption (600–900 nm) with distinct PA and therapeutic activation under PW and CW irradiation. Comparative studies confirmed PA signal generation under PW excitation with a negligible temperature rise, whereas CW excitation triggered ROS production and significant singlet oxygen generation. Cellular studies using MCF‐7 cells showed markedly reduced viability under CW excitation compared with that under PW excitation, validating selective phototherapy activation. In vivo PAI tracked the tumor accumulation of UCNPs‐DI over 24 h, peaking at 12 h post‐injection, before gradual clearance. This enables precise real‐time tumor visualization without phototoxicity during imaging. The orthogonal design minimizes unintended tissue damage, demonstrating safe long‐term diagnostic capability and effective on‐demand phototherapy.

In addition to remotely modulating PA contrast, light‐controlled drug release from theragnostic PA drug carriers enables localized delivery of therapeutic agents to target lesions. This approach often utilizes photoactivatable valve‐like molecular structures that can reversibly control the size of drug release channels. These structures act as gatekeepers, allowing precise spatiotemporal control of drug release in response to light stimuli. Li et al. developed a targeted PA‐FL bimodal imaging‐guided chemo‐photothermal therapy system by incorporating Cys‐P‐RGD, a reversible thermosensitive polypeptide‐engineered valve, into Ag_2_S quantum dot‐coated dendritic mesoporous silica (Ag_2_S@M‐P‐RGD) to control the release of the loaded anticancer doxorubicin (DOX).^[103]^ NIR irradiation of the quantum dots induces a temperature increase, unfolding the coil‐shaped polypeptide structure and promoting drug release. Repeated irradiation cycles resulted in a stepwise drug release profile. Multimodal imaging revealed the strong tumor targeting by the Ag_2_S@M/D‐P‐RGD nanocarriers, which peaked at 12 h post‐injection. The nanocarriers demonstrated effective photothermal conversion, raising tumor temperatures to 53°C under NIR irradiation. Combined chemo‐photothermal therapy using multiple short‐term laser exposures achieved superior tumor inhibition while minimizing skin damage compared with single long‐term irradiation.

Similarly, Yang et al. achieved precise kinetic control of drug release and photothermal therapy (PTT) using light by combining polypyrrole nanoparticles with an upper critical solution temperature (UCST) polymer template (CPT@DOX‐UCST/PPy).^[104]^ The size of the nanoparticles reversibly dilates (∼106 nm at 60°C) and shrinks (∼70 nm at 25°C) in response to the changes in temperature. The external photothermal trigger with a CW 808 nm laser enabled photothermal tumor ablation and the systemic initiation of chemotherapy through two mechanisms: the release of the loaded drug and the activation of the loaded doxorubicin (DOX) prodrug into its active form. PAI with a pulsed 808 nm laser revealed the accumulation and retention of theranostic particles within the 4T1 tumor, providing precise guidance regarding the location for the activation of the combined therapeutic treatment. The CPT@DOX‐UCST/PPy treatment combined with NIR laser irradiation significantly improved tumor growth inhibition and extended the survival time of the mice compared to the other treatment groups.

#### 3.1.2 Temperature stimuli

**FIGURE 4:**
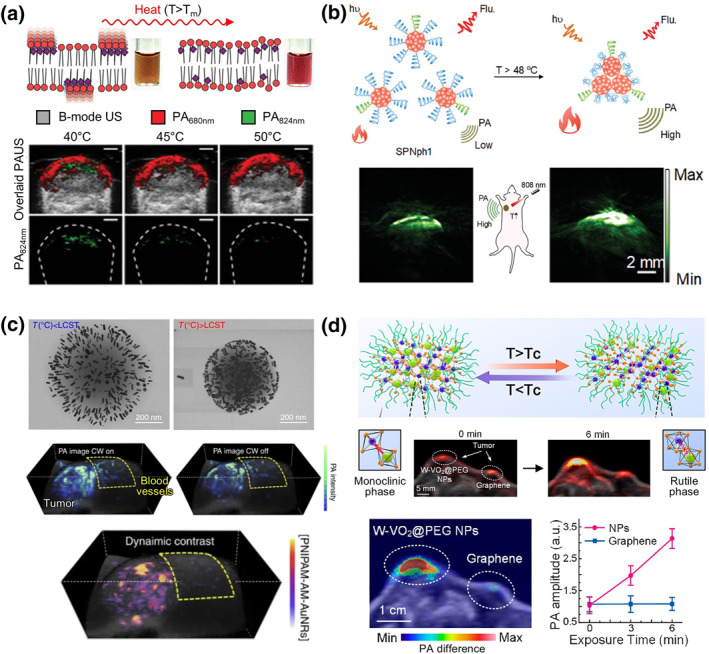
Temperature‐responsive contrast agents. (a) Reversibly switchable J‐aggregating nanovesicles (JNPs). The porphyrin walls of the nanovesicle rearrange with temperature changes, shifting peak absorption from 824 nm (dark brown) to 750 nm (bright pink); thermochromic PA contrast was demonstrated in vivo to report the intratumoral temperature during thermal therapy. Reproduced from ref. ^[105]^ with permission from American Chemical Society, copyright 2014. (b) Thermoresponsive semiconducting polymer nanoparticles (SPNs). At 48°C, reversible phase separation of surface brushes induces nanoparticle aggregation and enhances PA signal intensity; tumor‐targeted SPNph1 accumulates in 4T1 tumors and achieves a 43% signal‐to‐background ratio (SBR) increase upon NIR heating. Reproduced from ref. ^[106]^ with permission from John Wiley & Sons, copyright 2019. (c) Thermo‐responsive PNIPAM nanogel–based nanoparticles. Temperature‐triggered clustering and de‐swelling yield up to a sevenfold PA contrast enhancement upon laser heating, enabling dynamic contrast–enhanced PAI that suppresses endogenous background and highlights intratumoral uptake in murine prostate cancer xenografts. Reproduced from ref. ^[107]^ with permission from Springer Nature, copyright 2017. (d) Thermally reversible W‐VO_2_@PEG nanoprobes. PEG‐coated, tungsten‐doped VO_2_ switches between monoclinic (<38°C) and rutile (>38°C) phases to switch on NIR‐II absorption for deep tissue imaging; in EMT6 tumor–bearing mice, 980 nm laser irradiation (0.5 W/cm^2^) heated tumors to 43°C in 5 min, producing enhanced PA contrast for improved differential imaging. Reproduced from ref. ^[108]^ with permission from American Chemical Society, copyright 2022. NIR, near‐infrared, PA, photoacoustic; PAI, photoacoustic imaging; PEG, polyethylene glycol.

Ng et al. were the first to introduce thermochromic nanovesicles for in vivo PAI applications, to develop reversibly switchable temperature‐dependent J‐aggregating nanoparticles (JNPs, Figure 4a).^[105]^ Inspired by the optimization of light absorption by photosynthetic microorganisms, they designed light‐harvesting porphyrin nanovesicles that changed arrangements within aggregates when heated, consequently changing their color from dark brown (824 nm peak absorption) to bright pink (750 nm peak absorption). The JNPs demonstrated highly repeatable thermochromic responses in reciprocated thermal cycle tests. Upon heating the JNP gel phantom, its 750 nm/824 nm dual‐wavelength PA contrast revealed a transient heat flux map. In vivo demonstration of JNP‐based intratumoral PA temperature sensing showed a 2.7‐fold decrease in the intensity of the 824 nm PA when the tumor temperature exceeded 44°C. These results confirm the potential of JNPs as thermographic imaging agents for various temperature‐related applications.

Given that PA agent signal generation can be regulated through external temperature control, setting the temperature to maximize PA signal generation facilitates an increase in the signal‐to‐background ratio (SBR) and improves the molecular specificity of PA images. Cui et al. synthesized self‐assembled thermoresponsive SPNs conjugated with PDMA‐r‐HPA brushes (Figure 4b).^[106]^ Owing to their low critical solution temperature (LCST) of 48°C, the brushes undergo reversible phase separation upon heating, promoting the aggregation of SPN, inflating the particle size, and ultimately enhancing the PA signal intensity. Leveraging the high photothermal conversion efficiency (34.9%) of SPNph1, continuous 808 nm laser irradiation heated the SPN to 50°C, consistent with its LCST. At this temperature, the PA intensity of SPNph1 increased 1.57‐fold compared to that at 25°C, demonstrating temperature‐dependent signal enhancement. In vivo PAI of 4T1 xenograft tumors revealed that SPNph1 accumulated in tumors over 24 h post‐injection, with PA signals 10% higher than those in the liver. After 1 min of 808 nm laser irradiation, the PA signal‐to‐noise ratio (SNR) in SPNph1‐treated tumors increased from 4.16 to 6.12, representing a 43% SBR enhancement. These results demonstrate the potential of these novel thermoresponsive PA nanoagents for high‐contrast tumor imaging.

Pursuing a similar goal of improving SBR, Chen et al. focused on distinguishing the exogenous PA contrast from its dynamic fluctuation following an externally modulated stimulus (Figure 4c).^[107]^ Thermo‐responsive PNIPAM nanoparticles were synthesized by loading AuNRs and CuS nanospheres onto PNIPAM nanogels. The PNIPAM nanoparticles achieved deep tissue penetration owing to their strong absorption in the NIR‐I and NIR‐II windows, while serving as photothermal therapeutics, surpassing the capabilities of conventional triggerable fluorophores. The PA intensity of the PNIPAM‐AuNR nanoconstructs was 3.5 times higher than that of the pure AuNR solutions, with significant enhancements attributed to clustering and de‐swelling effects as the temperature varied around their LCST. In an ex vivo animal injected with PNIPAM‐CuS nanoconstructs, the subtraction of two PA images obtained with the heating laser on and off resulted in a seven‐fold increase in the imaging contrast, owing to the effective suppression of the background PA contrasts unrelated to the temperature changes. Such dynamic contrast‐enhanced PAI was replicated in a murine prostate cancer xenograft model, showing a 5.3‐fold increase in the contrast 24 h after the injection of PNIPAM‐AuNRs, indicating their accumulation in the tumor and clearance from circulation.

Despite their outstanding performance, the practical utility of thermoresponsive PA nanoagents is hindered by their transition temperature, which for most reported PA NPs is generally much higher than human body temperature, posing a potential risk of thermal damage. Moreover, the assembly or aggregation‐dependent applications generally exhibit slow response speeds and poor temperature sensitivities, limiting their dynamic probing capabilities. Li et al. addressed these issues by proposing polyethylene glycol (PEG)‐coated tungsten‐doped vanadium dioxide (W‐VO_2_@PEG) as a novel nanoprobe that is triggered at the human body temperature (Figure 4d).^[108]^ The insulator‐to‐metal phase transition within the complex occurs together with the thermally‐reversible transformation between the rutile phase (above 38°C) and the monoclinic phase (below 38°C), enabling the instant “switching on” of optical absorption in the NIR‐II region when the temperature exceeds the critical point. Additionally, the NIR‐II (1000–1700 nm) absorption characteristics of the complex allow for a much higher maximum permissible laser exposure and reduced scattering compared to NIR‐I (700–950 nm), facilitating contrast‐enhanced PAI of deep tissues. In vitro chicken tissue phantom tests demonstrated that a tumor‐mimicking VO_2_@PEG NP‐loaded agar sample could be detected beneath the chicken breast tissue (1.5 cm). The PA signal amplification ratio between 35°C and 45°C was 300 ± 41%, demonstrating significant temperature‐dependent signal enhancement. In vivo experiments on EMT6 tumor‐bearing mice demonstrated that 980 nm laser irradiation (0.5 W/cm^2^) could remotely heat tumors to 43°C within 5 min. W‐VO_2_@PEG NP‐injected tumors displayed PA contrast, which was further enhanced by 980 nm laser exposure. This temperature triggered an improvement in tumor visualization through differential imaging. Extending the work of Li et al., Sun et al. repurposed W‐VO_2_@PEG as a PA nanothermometer (NThem) that linearly responds to the human body temperature range, with an accuracy of 0.3°C.^[109]^ NThems enable real‐time three‐dimensional (3D) temperature monitoring of EMT6 tumors during PTT, demonstrating their effectiveness for precise and quantitative guidance in cancer treatment, enhancing safety and efficacy.

In a similar temperature range, Ma et al. repurposed commercially‐available thermochromic materials encapsulated by a biocompatible transparent polymer alginate shell, named as reversibly switchable thermochromic microcapsules (RSTM).^[110]^ The color changes as the red or black chromic materials dissolve in the solid‐state solvent when the temperature is below the crystallization temperature (25°C). Upon heating up to 37°C, the solvent transforms into a liquid state, and the dye dissociates, resulting in a recoverable loss or decrease in color. Specifically, the absorption peaks of black‐to‐colorless RSTM at 460 and 590 nm faded upon heating, decreasing the absorbance by 53%. Furthermore, an RSTM‐loaded Matrigel implant in the mouse leg was imaged via PAI during repeated heating and cooling between 20°C and 40°C, and the resultant differential image displayed effective removal of non‐switching background signals and enhancement in the RSTM contrast. Differential imaging effectively mitigated the challenge of decreasing the PA signal amplitude due to optical attenuation, validating imaging capabilities up to 13‐mm depth in vivo.

In contrast to the traditional photosensitizers that generate PA signals through direct laser excitation, light‐activated sonophores offer a promising approach for potentially generating higher PA signal enhancement. Wilson et al. pioneered a novel approach for enhancing PA and US imaging by developing photoacoustic nanodroplets (PAnDs).^[111]^ These PAnDs consist of liquid PFC nanodroplets that encapsulate photothermal nanoparticles. Upon pulsed laser irradiation, the nanoparticles rapidly heat up, triggering the vaporization of the PFC core. This process creates microbubbles, instantly activating a strong PA signal together with enhanced US contrast.[^112^, ^113^, ^114^] Using organic semiconducting PA nanodroplets encapsulated together with PDT photosensitizer (PS‐PDI‐PAnDs), Tang et al. broadened the range of nanodroplet functionalities toward cancer therapy.^[115]^ Even in hypoxic peritumoral environments, oxygen‐rich PFCs can supply the oxygen necessary for efficient photodynamic ROS generation. Activated PS‐PDI‐PAnDs were visible in PAI and USI, and their in vivo PA/US/FL multimodal imaging comprehensively indicated their accumulation in the tumor, reaching the maximum concentration at 24‐h post‐injection. Photodynamic therapy was induced using continuous 671 nm laser irradiation of the tumor, resulting in enhanced tumor oxygen levels compared to the conventional PDT approaches. Moreover, PTT and PDT can be simultaneously performed, with this synergistic approach achieving more effective tumor eradication compared to the PTT only‐ or PDT only treatments.

#### 3.1.3 Ultrasound stimuli

NIR stimuli are predominantly utilized as activation mechanisms to photochemically or photothermally trigger the responsive PA contrast agents. However, targeting deep‐seated tumors with light‐based triggers remains challenging because of the limited tissue penetration depth. By contrast, acoustic waves exhibit superior tissue penetration with less scattering and also offer cost‐effectiveness and biosafety. Ultrasound (US) has been extensively employed in diagnostic imaging and therapy, and the advent of sonosensitive nanoagents is poised to enhance drug delivery and chemotherapeutic efficacy.

**FIGURE 5:**
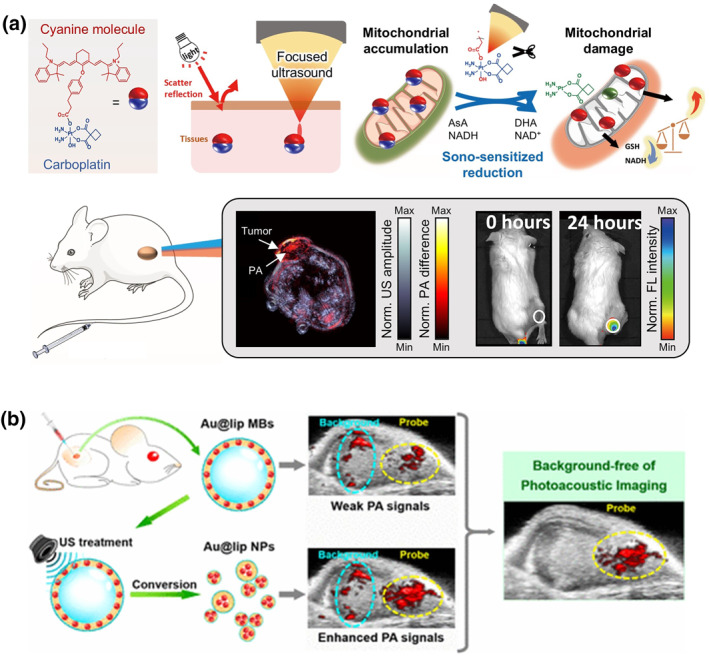
Ultrasound‐responsive contrast agents. (a) Sonosensitive nanodrug “cyanoplatin,” which integrates a reductively activatable Pt(IV) carboplatin prodrug with a sonosensitive cyanine dye (IR780). Sonoexcitation triggers reduction of Pt(IV) to cytotoxic Pt(II), enhancing anticancer efficacy; in vivo PA and FL imaging trace its biodistribution, showing increased tumor accumulation. Reproduced from ref. ^[116]^ with permission from the American Association for the Advancement of Science, copyright 2023. (b) Gold nanoparticle–lipid shell microbubbles (Au@lip MBs). Insonification ruptures the microbubbles into nanoscale Au@lip aggregates that generate strong NIR PA signals; subtraction of pre‐ and post‐burst PA images suppresses endogenous background signals, revealing molecularly specific distribution in CT26 tumors. Reproduced from ref. ^[117]^ with permission from American Chemical Society, copyright 2019. FL, fluorescence; MBs, Microbubbles; NIR, near‐infrared; PA, photoacoustic.

Liu et al. devised an ultrasound‐activatable prodrug that initiated localized chemotherapy using acoustic radiation (Figure 5a).^[116]^ This sonosensitive nanodrug, termed “cyanoplatin”, combines a reductive Pt(IV)‐based prodrug (carboplatin) and sonosensitive heptamethine cyanine (IR780). The cyanine counterpart serves to generate optical FL and PA contrast upon laser irradiation and to induce the reduction of the Pt(IV) core into activated cytotoxic Pt(II) upon sono‐excitation. Based on the investigation of its dual activation pathways, sono‐excitation enhanced the cyanoplatin oxidation capability through singlet oxygen generation and sono‐sensitized electron transfer, potentially augmenting its anticancer chemotherapeutic efficacy. Comparative analysis of photo‐ and sono‐activation of cyanoplatin through varying thicknesses of chicken breast tissue revealed the superior efficacy of sono‐activation, which maintained significant activity even at a depth of 20 mm, whereas photoactivation became ineffective beyond 10 mm. This demonstrates the enhanced tissue penetration capability of US for deep‐seated prodrug activation compared with light‐based methods. The biodistribution of injected cyanoplatin was tracked via longitudinal PACT and FLI, both of which showed maximum tumor accumulation at 12 h post‐injection. Multimodal PACT/FLI guidance enables precise spatiotemporally controlled FUS theranostics, facilitating accurate tumor treatment with minimal adverse effects.

Microbubbles (MBs) are gas‐filled microspheres that are typically used as contrast agents in clinical USI. Their inert gas cores are highly reflective and hyperechogenic in USI. Furthermore, the MBs can be cavitated because they are compressible by external pressure, and insonification above the ultimate limit of acoustic pressure may induce the microbubbles to burst and fragment into even smaller pieces. Meng et al. applied this concept to achieve background‐free in vivo PAI by manipulating the PA contrast of gold nanoparticle‐lipid shell MBs (Au@lip MBs) via bursting (Figure 5b).^[117]^ Au NPs within MBs are sparsely separated within a thin lipid layer in their complete shape, exhibiting a relatively low NIR PA contrast arising from their low NIR absorbance owing to low local concentration. US pulses trigger MB rupture, forming nanoscale Au@lip aggregates that produce stronger NIR PA signals due to red‐shifted SPR absorption. Because Au@lip MBs are uniquely sensitive to US exposure, subtracting the pre‐ and post‐burst PA signals eliminates the background noise from endogenous chromophores, revealing only the Au@lip MB distribution within the PAI. Au@lip MBs in CT26 tumors of mice showed significant enhancement of PA signals after US bursting, whereas PA signals from control chromophores (CuS NPs) were fully recovered in the reconstructed image. Subtractive imaging with the Au@lip MB effectively eliminated the false PA signal rise caused by cancerous vascular proliferation and isolated the accumulation effect of the agent. Moreover, Erlotinib (EB)‐treated tumors showed significantly higher Au@lip MB accumulation than the untreated tumors, suggesting enhanced intratumoral penetration due to EB‐induced vascular normalization.

#### 3.1.4 Electromagnetic field stimuli

Magneto‐motive photoacoustic (mmPA) imaging, developed by Jin et al., utilizes magnetic nanoparticle (MNP)‐gold core‐shell NPs with strong NIR PA and MRI responses.^[118]^ This technique is applied to a pulsed magnetic field during imaging, inducing NP movement followed by the return of the NPs after the field is turned off. By detecting coherent motion in the PA image sequences, mmPA isolates MNP signals and rejects nonmagnetic background noise, thereby enhancing contrast specificity. The synthesized NPs (<25 nm diameter, 3–4 nm gold shells) exhibited enhanced SPR peaks in the NIR band (660–900 nm), appearing 20 dB brighter in the PA images than in the MNP core. The magnetic properties were largely preserved after the gold coating. The MNP‐gold core‐shell NPs showed superior photostability compared with AuNRs and nanocages under high fluence irradiation (10–15 mJ/cm^2^). For mmPA processing, PAI frames were acquired over a 10‐s interval with equal magnetic pulse‐on and pulse‐off periods. The NP displacement was tracked using a speckle tracking algorithm and converted to velocity profiles. The resulting mmPA image selectively visualized the MNP‐gold hybrid NPs while suppressing the signals from the non‐magnetic AuNRs.

**FIGURE 6:**
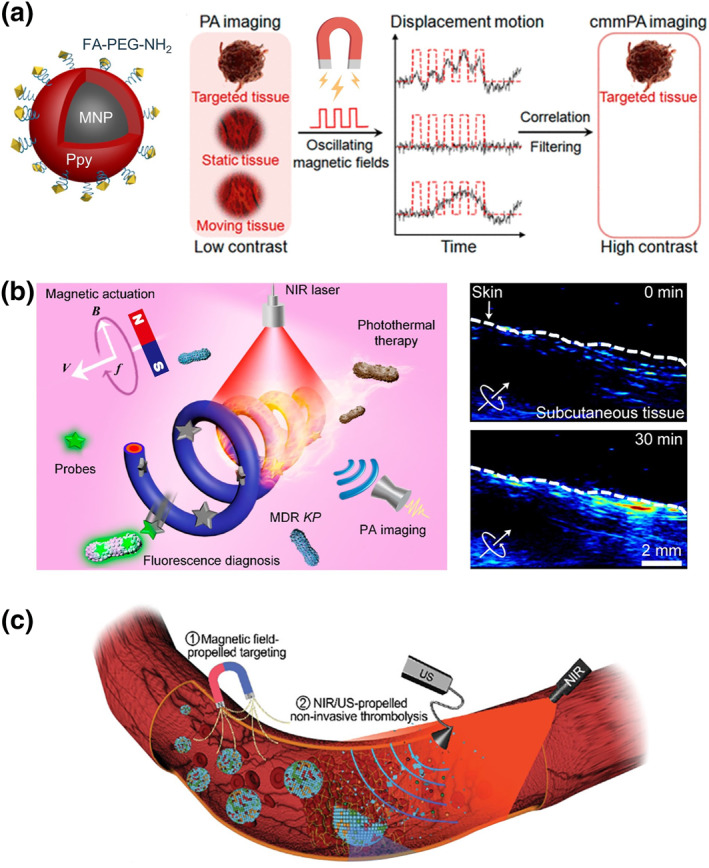
Electromagnetic‐responsive contrast agents. (a) Magnetic nanoparticle (MNP)‐polypyrrole (Ppy) core‐shell nanoparticles for magneto‐motive photoacoustic imaging. Pulsed magnetism induces transient movement of MNPs from PAI, where filtering the motions coherent with the oscillating magnetic pulses separates the MNP‐specific contrast in the targeted tissue. Reproduced from ref. ^[119]^ with permission from American Chemical Society, copyright 2015. (b) Helix‐shaped magnetized Spirulina (MSP) as an ingestible magnetic microswimmer. Rich PA contrast from polydopamine coating and inherent red FL contrast from algae at 552 nm enable noninvasive tracking in real‐time PA and FL imaging. Active localization of microswimmer mouse subcutaneous bacterial infection lesion is demonstrated by significant PA signal enhancement after magnetic actuation. Reproduced from ref. ^[120]^ with permission from American Chemical Society, copyright 2020. (c) Novel thrombolytic therapy using magnetic‐propelled nanomotors. Magnetically guided iron oxide nanomotors loaded with perfluorohexane and urokinase generate gas bubbles under NIR/US stimulation, inducing cavitation for enhanced deep thrombus penetration and targeted thrombolysis. Real‐time PAI, USI, and color Doppler USI enable localization and monitoring, with PAI demonstrating a 4.7‐fold signal enhancement at the thrombus site. Reproduced from ref. ^[121]^ with permission from John Wiley & Sons, copyright 2023. FL, fluorescence; NIR, near‐infrared; PA, photoacoustic; PAI, photoacoustic imaging; US, Ultrasound.

To accelerate potential clinical translation, Li et al. developed ∼50 nm MNP‐PPy core‐shell nanoparticles with enhanced photostability and implemented them in a next‐generation imaging strategy called cyclic mmPA, which greatly improved the robustness of displacement tracking (Figure 6a).^[119]^ The MNP‐PPy nanoparticles demonstrated 10 times stronger PA signal at 800 nm compared to MNPs alone and superior photostability over AuNRs under high‐intensity laser irradiation. Cyclic mmPA images demonstrated an exceptional capability for selectively visualizing magnetically tagged cancer cells in vivo, achieving sub‐micrometer displacement detection and robust signal isolation even with significant background motion. By utilizing multiple magnetic excitation cycles and time coherence‐based thresholding, the technique successfully distinguished the actual magneto‐motion from complex physiological movements, enabling the clear delineation of tagged cells beneath the skin tissue. This method shows high sensitivity and the potential for advanced molecular imaging applications, with minimum detectable displacements as low as 0.8 μm.

The use of magnetic fields for remote control of micromotors and microrobots in living organisms has been extensively studied in ingestible medical device engineering. Xie et al. reported a magnetic microswimmer fabricated from a polydopamine (PDA)‐layered magnetized Spirulina (MSP) matrix consisting of helix‐shaped algae coated with magnetite nanoparticles (Figure 6b).^[120]^ These microswimmers provide dual imaging capabilities: real‐time tracking through the strong PA contrast generated by the PDA coating and inherent red FL contrast from the MSP core when excited at 552 nm. From in vivo experiments, the microswimmers were effectively localized within a mouse model of subcutaneous bacterial infection, achieving significant PA signal enhancement after magnetic actuation. Time‐lapse PA images indicated the subcutaneous accumulation of the microswimmers, highlighting their potential for targeted diagnosis and treatment. Furthermore, PTT using PDA led to a substantial reduction in the number of bacterial colonies and accelerated healing, with complete epithelialization observed by day 12.

A treatment plan involving precisely designed multi‐step triggered actuation can achieve optimal therapeutic efficacy. Traditional clinical thrombolytic strategies for thromboembolism are often hindered by poor medication penetration into the thrombi. Ruan et al. addressed this limitation by developing a novel nanomotor for a three‐step propelled cascade thrombolytic therapy (Figure 6c).^[121]^ These nanomotors, composed of magnetic iron oxide, NIR/US‐triggered perfluorohexane (PFH), and the thrombolytic agent urokinase, can be magnetically guided to cluster near the target thrombi. Subsequently, local photothermal or US stimuli triggers the liquid PFH to convert into gas bubbles, creating a cavitation effect that enhances penetration into the deep thrombus and liquifies the clot. Finally, urokinase is released from the collapsed nanomotors to achieve pharmaceutical thrombolysis. Nanomotors can be localized in real‐time and monitored throughout the course of the treatment via a combination of PAI, USI, and Color Doppler USI, enhancing the precision and effectiveness of the treatment. PAI enabled high‐resolution thrombus monitoring, revealing a 4.7‐fold increase in the signal intensity at the thrombus site compared with normal blood vessels. Concurrently, color Doppler US imaging confirmed thrombus formation by effectively differentiating veins and arteries based on the flow direction and spectral Doppler patterns, with veins showing gentle trends and arteries exhibiting high cusps and intermittent spectra.

Table 1 lists a comprehensive overview of the methodologies and key findings of the selected studies.

**TABLE 1:** Summary of externally triggered photoacoustic contrast agents.

Ref	Stimuli	Name	Condition	Switching mechanism	Multimodality	Peak absorption wavelength (nm)	Absorbance ratio (on/off)	Imaging wavelength (nm)	PA signal switching ratio	Target application
^[96]^	NIR	DTE‐DPA‐SCP	UV (on), 660 nm (off)	Light‐responsive molecular ring transform	PA, FL	635 (on)	∼12	650	∼10	Pre/intraoperative tumor imaging
^[97]^	NIR	mDrBphP‐PCMm/F469W	630 nm (on), 780 nm (off)	Bacterial phytochromic protein	PA	780 (on)	15.99	780	∼4	Tumor monitoring
^[98]^	NIR	GLANCE	815 nm	Gas‐generating laser‐activatable nanorods	PA, CEUS	818 (on)	8.3	815	∼7.5	Tumor monitoring
^[99]^	NIR	UCNPs‐DI	980 nm (PW/CW)	Switchable upconversion nanoparticles	PA, PDT	675	n.a.	980	∼16.2	Tumor monitoring and treatment
^[103]^	NIR	Ag_2_S@M/D‐P‐RGD	808 nm (CW)	Polypeptide‐engineered valve	PA, FL, PTT, ChT	Undefinable	n.a.	744	n.a.	Tumor monitoring and treatment
^[104]^	NIR	CPT@DOX‐UCST/PPy	808 nm (CW)	Reversible swelling	PA, PTT, ChT	Undefinable	n.a.	808	∼10.6	Tumor monitoring and treatment
^[105]^	Temperature	Stimuli‐responsive nanoswitch	41–44°C	Reversible coherent dye J‐aggregation	PA	750/824	2.7	824	2.7	Tumor imaging
^[106]^	Temperature	PDMA‐r‐HPA	48°C	Thermoresponsive polymer brush	PA	∼700	n.a.	680	1.37	Tumor imaging
^[107]^	Temperature	PNIPAM nanoconstructs	32–37°C	Reversible swelling hydrogel	PA	760	n.a.	780	1.7 (ex vivo)	Tumor imaging
^[108]^	Temperature	W‐VO2@PEG	41.1°C	Thermochromic VO_2_ insulator‐to‐metal phase transition	PA	∼1100	∼4	1064	3.6	Tumor imaging
^[109]^	Temperature		39.5°C		PA	1150	∼2	1064	∼2	Tumor imaging
^[110]^	Temperature	RSTM	20–40°C	Alginate‐coated thermochromes	PA	525 (20°C)	10	532	n.a.	In vivo implant imaging
^[115]^	Temperature	PS‐PDI‐PAnD	37°C	Encapsulated PFC droplet with photoabsorber	PA, FL, US, PTT, PDT	671	n.a.	850	n.a.	Tumor monitoring and treatment
^[116]^	Ultrasound	Cyanoplatin	1.75 MHz, 4 W	Sonoactivation of carboplatin counterpart	NIR, PA, US	780	n.a.	780	n.a.	Tumor monitoring and treatment
^[117]^	Ultrasound	Au@lip MBs	20 kHz, 10 W	Microbubble US burst into Au NPs	PA/US	300–800	∼3	710	∼3.5	Tumor imaging
^[118]^	Electromagnetic	MNP‐Au	0.7 T	Iron oxide‐gold core‐shell	mmPA	∼800	n.a.	720	20 dB	Phantom imaging
^[119]^	Electromagnetic	Iron oxide–PPy	0.7 T	Iron oxide‐Ppy core‐shell	mmPA	800	∼4.8	800	∼10 (vs. MNPs)	Tumor imaging
^[120]^	Electromagnetic	Magnetize spirulina	8.0 mT (rotating)	Magnetic actuation of helical nanostructure	PA, FL, PTT	808	n.a.	800	n.a.	Tumor monitoring and treatment
^[121]^	NIR, ultrasound, electromagnetic	Liposome nanomotor	n.a.	Magnetic propelled targeting	PA, US, ChT	275 nm	n.a.	685	4.7 (vs. Vein)	Thrombolysis treatment

Abbreviations: CW, continuous wave; FL, fluorescence; GLANCE, gas‐generating laser‐activatable nanorods for contrast enhancement; mmPA, magneto‐motive photoacoustic; NIR, near‐infrared; PA, photoacoustic; PDT, Photodynamic therapy; PPy, polypyrrole; PTT, Photothermal therapy; PW, pulsed wave; US, ultrasound.

### 3.2 Microenvironment‐triggered photoacoustic contrast agents

The tumor microenvironment differs markedly from normal tissues, with acidic pH, elevated ROS, hypoxia, abundant glutathione (GSH), and overexpressed enzymes driven by rapid tumor growth and metabolism. These unique features have motivated the development of microenvironment‐responsive photoacoustic contrast agents, which are selectively activated in tumor tissues. By generating strong, tumor‐specific signals, these agents improve diagnostic accuracy by reducing background interference and enhancing SNR.

#### 3.2.1 Hypoxia responsive

Hypoxia is a common feature of solid tumors and plays a critical role in cancer progression and prognosis. In the tumor microenvironment (TME), hypoxia induces overexpression of various reductases. This phenomenon is a part of the cellular adaptation mechanism for survival under oxygen‐deficient conditions. In the absence of oxygen as a terminal electron acceptor, cells activate alternative reduction pathways to maintain metabolic balance. This leads to increased expression of specific reductases, such as nitroreductase (NTR) and azoreductase, which serve as crucial biological markers of hypoxia. These hypoxic enzyme‐mediated reactions can be utilized to enhance the specificity of hypoxia‐responsive PA contrast agents.

**FIGURE 7:**
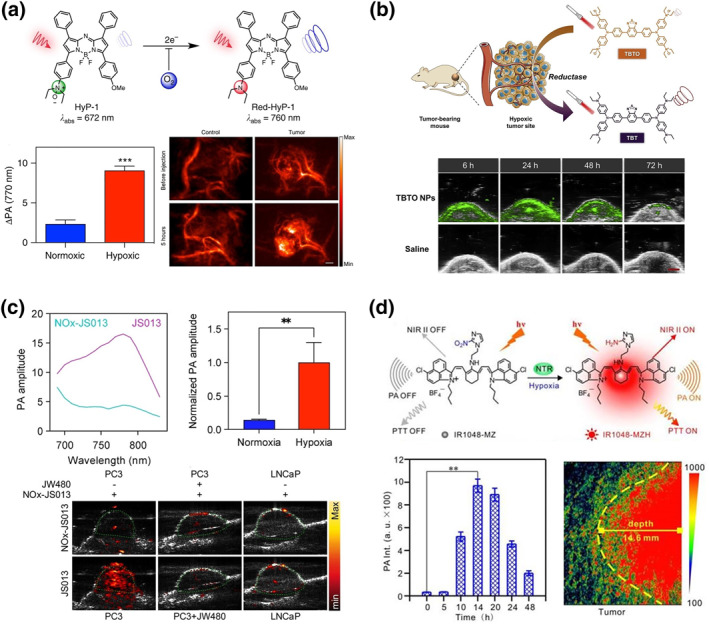
Hypoxia‐responsive contrast agents. (a) Hypoxia‐responsive PA probe featuring a bioreducible N‐oxide trigger (HyP‐1). Upon reduction under hypoxia, its PA absorbance shifts from 670 to 760 nm, resulting in a fourfold PA signal enhancement at 770 nm. In vivo, this results in a contrast increase by 20.5 times and enables 3D visualization of tumor hypoxia. Reproduced from ref. ^[122]^ with permission from Springer Nature, copyright 2017. (b) TBTO with four diethylamino N‐oxide groups that is reduced to the D‐A‐D molecule TBT under hypoxia, activating the TICT mechanism to generate strong PA signals at 900 nm. TBTO shows excellent biocompatibility and produces tumor‐selective PA contrast in vivo, persisting for up to 48 h. Reproduced from ref. ^[123]^ with permission from Elsevier, copyright 2021. (c) The Aza‐BODIPY–based PA probe NOx‐JS013 exhibits a 7.1‐fold signal increase at 770 nm upon hypoxic reduction and covalently targets NCEH1 for enhanced specificity. In prostate cancer models, NOx‐JS013 distinguished aggressive PC3 from non‐aggressive LNCaP tumors with significant in vivo PA contrast and an 8.2‐fold higher ex vivo fluorescence ratio, confirming its tumor specificity. Reproduced from ref. ^[124]^ with permission from John Wiley & Sons, copyright 2024. (d) IR1048‐MZ by conjugating a nitroimidazole trigger to IR1048 so that nitroreductase‐mediated reduction under hypoxia restores its PA signal and activates photothermal therapy. In A549 tumor‐bearing mice, IR1048‐MZ enabled clear 3D PAI of hypoxic regions 10 h post‐injection with up to 14.6 ± 0.2 mm penetration depth. Reproduced from ref. ^[125]^ with permission from Ivyspring International Publisher, copyright 2018. PA, photoacoustic; PAI, photoacoustic imaging; TBTO, triphenylamine‐benzothiadiazole‐triphenylamine derivative featuring four diethylamino N‐oxide groups, TICT, twisted intramolecular charge transfer.

Knox et al. synthesized a hypoxia‐responsive PA probe (HyP‐1), characterized by a bioreducible N‐oxide trigger that is activated under oxygen‐deficient conditions (Figure 7a).^[122]^ Under hypoxia, heme proteins such as CYP450 enzymes reduce N‐oxide to aniline, shifting its absorbance peak from 670 to 760 nm owing to enhanced electron donation. Under hypoxia conditions, HyP‐1 exhibited a fourfold increase in the PA signal at 770 nm. In tumor‐bearing mice, PAI demonstrated a significant PA signal enhancement in tumor tissues, with an average increase of 20.5. The reconstructed 3D PA images showed high‐signal regions within the tumors, confirming the ability of the probe to detect hypoxia.

Li et al. designed a hypoxia‐activated probe, TBTO (triphenylamine‐benzothiadiazole‐triphenylamine derivative featuring four diethylamino N‐oxide groups), which generated strong and selective PA signals at hypoxic tumor sites (Figure 7b).^[123]^ TBTO undergoes bioreduction in hypoxic tumor microenvironments to form TBT, a molecule with a D‐A‐D structure through a twisted intramolecular charge transfer mechanism, emitting strong PA signals upon photoexcitation before returning to the ground state. TBTO exhibits excellent biocompatibility and high reactivity in hypoxic tumor microenvironments. After conversion to TBT, an intense PA signal was observed at 900 nm, which persisted for up to 48 h in tumor regions of the mouse models.

Song et al. developed a HyP‐1 that incorporated enzyme targeting to enhance specificity in aggressive TMEs (Figure 7c).^[124]^ NOx‐JS013 is a PAI probe that noninvasively detects hypoxia and features high photostability and strong absorptivity in the NIR window. The probe integrates Aza‐BODIPY‐based Hyp‐1 to produce a strong PA signal at 770 nm. NOx‐JS013 showed a 7.1‐fold increase in the PA signal at 770 nm under hypoxia compared to normoxia, indicating efficient activation in the presence of CYP enzymes. The probe was further modified with a moiety for selective covalent binding to NCEH1, an enzyme that is overexpressed in aggressive prostate tumors. NOx‐JS013 showed significant differences in the PA signals between non‐aggressive (LNCaP) and aggressive (PC3) prostate cancer models. Tumor specificity was further confirmed by ex vivo FL analysis, which showed an 8.2‐fold higher JS013/NOx‐JS013 ratio in the tumors compared to lungs, underscoring the high tumor selectivity of Nox‐JS013. The dual‐responsive design effectively reduced background signals and enhanced sensitivity to aggressive tumors.

Meng et al. designed a hypoxia‐activated PA probe, IR1048‐MZ, that responds to NTR, a biomarker of hypoxia (Figure 7d).^[125]^ Under hypoxic conditions, NTR uses NADH as an electron donor to reduce nitro compounds to amines. IR1048‐MZ was constructed by conjugating a nitroimidazole group (MZ) with IR1048. The electron‐withdrawing MZ group lowered the electron density of IR1048, resulting in reduced absorbance and weak PA signals. Upon reduction to the amine group, the PA signal of IR1048‐MZ was restored, enabling selective PAI. In A549 cancer mouse models, IR1048‐MZ enabled 3D PA signal monitoring, with a clear distinction in the tumor region observed 10 h post‐injection. The PAI exhibited a maximum penetration depth of 14.6 ± 0.2 mm, demonstrating the capability of the probe for deep‐tissue imaging. Additionally, IR1048‐MZ functioned as a photothermal agent under hypoxic conditions, enabling tumor‐specific therapy. Selectivity was validated using a reductase inhibitor.

#### 3.2.2 pH responsive

pH is a crucial biomarker of various pathological conditions, including cancer, inflammation, and ischemia. Tumor microenvironments exhibit acidic pH owing to hypoxia and high metabolic rates, making pH‐responsive PA probes valuable tools for selective tumor imaging. These probes leverage pH‐dependent changes in optical properties, aggregation behavior, and structural modifications to enhance the PA signal contrast and specificity.

**FIGURE 8:**
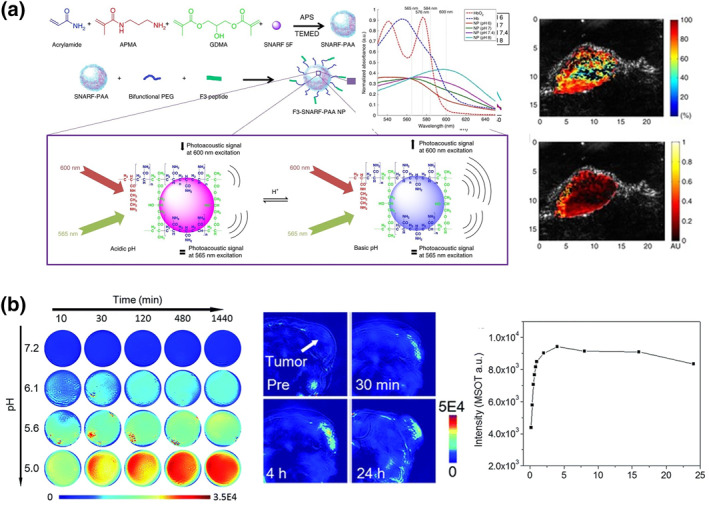
pH‐responsive contrast agents. (a) SNARF‐PAA nanoparticles enabled pH‐responsive PAI in the pH range of 5.8–7.8. Quad‐wavelength imaging minimized hemoglobin interference, allowing precise mapping of tumor acidosis and hypoxia. Reproduced from ref. ^[126]^, with permission from Springer Nature, copyright 2017. (b) Prussian blue‐based hydrogels exhibited pH‐dependent photoacoustic imaging, showing distinct signal variations over time in tumor environments and across buffers with varying pH levels. These results confirm the agent’s suitability for dynamic monitoring in acidic microenvironments. Reproduced from ref. ^[127]^ with permission from Royal Society of Chemistry, copyright 2017. PAI, photoacoustic imaging; SNARF‐PAA, SNARF‐5F encapsulated polyacrylamide nanoparticles.

Various strategies have been employed to optimize signal contrast and biocompatibility of pH‐responsive PAI. Jo et al. developed SNARF‐5F encapsulated polyacrylamide nanoparticles (SNARF‐PAA NPs) as a pH‐responsive PAI contrast agent, which is formed under a pH range of 5.8–7.8 and modifies the surface to target tumors and avoid the immune system (Figure 8a).^[126]^ SNARF‐5F is a well‐known ratiometric pH indicator that exhibits pH‐dependent spectral shifts between 565 and 600 nm, enabling dual‐wavelength PA measurements. Quad‐wavelength PA measurement (565, 576, 584, 600 nm) ensured precise tumor pH mapping and address separating hemoglobin absorption in biological environments interfering under such band. In vivo PAI of tumor‐bearing mice demonstrated real‐time acidosis detection, with a significant contrast between the tumor and surrounding tissues. In addition, long‐term monitoring indicated stable probe retention and minimal degradation, supporting its potential for clinical translation.

A different approach was explored by Yang et al., who designed polyaniline‐bovine serum albumin (PANI‐BSA) nanoparticles to enhance the PA contrast in acidic environments.^[128]^ PANI, which shows poor solubility and a tendency to aggregate in aqueous condition, is stabilized via BSA conjugation, significantly improving its biocompatibility, aqueous dispersibility, and tumor specificity. The probe exhibited a linear PA intensity increase in the pH range of 5.0–6.8 under 800 nm excitation, demonstrating robust signal enhancement under tumor‐mimicking conditions. In vivo imaging confirmed clear tumor delineation with a high SNR, reinforcing its suitability for precise tumor acidity mapping. Moreover, the photostability and minimal cytotoxicity of PANI‐BSA ensures its reliability for extended imaging applications.

Guha et al. developed croconaine‐based liposomes (CrocRot) as a pH‐responsive PA contrast agents.^[129]^ Croconaine dyes undergo a pH‐induced absorption shift from 660 nm (basic) to 794 nm (acidic), enabling ratiometric PAI. However, to prevent aggregation and photochemical instability, croconaine was encapsulated in stealth liposomes (Croc‐SL) to enhance its biocompatibility and in vivo retention. PAI demonstration of Croc‐SLs exhibited robust pH differentiation, effective tissue acidosis detection, and stable photothermal conversion. In tumor‐bearing mouse models, CrocRot exhibited strong PA signals in acidic tumor regions in contrast to healthy tissues. Its dual imaging and therapeutic potentials further establish its potential as a multifunctional agent for tumor monitoring and treatment.

To further improve signal specificity, Chen et al. introduced HSA‐BPOx‐IR825 nanoprobes for ratiometric PA pH imaging.^[130]^ These self‐assembled nanoprobes integrate pH‐sensitive benzo‐a‐phenoxazine (BPOx) and pH‐invariant IR825, allowing precise dual‐wavelength detection (680 and 825 nm). Under acidic tumor conditions, the PA intensity at 680 nm increased significantly, whereas the reference signal at 825 nm remained stable, ensuring accurate pH quantification. In vivo studies demonstrated clear tumor‐specific accumulation with minimal background interference, validating the ability of the probe to provide spatially accurate acidity mapping. Additionally, prolonged imaging stability and minimal toxicity suggest the high potential of BPOx for future clinical applications in real‐time tumor diagnostics.

Advances in semiconducting nanomaterials have contributed to the development of highly sensitive PAI contrast agents. Semiconducting oligomer nanoparticles (SONs) have been engineered to amplify PA contrast in acidic environments.^[131]^ SONs composed of f‐DTS matrices doped with BODIPY dyes (pH‐BDP) exhibit tunable absorption between 621 and 750 nm, making them highly adaptable for pH‐responsive imaging. At pH 5.5, the PA intensity increased 3.44‐fold compared with that at neutral pH, indicating exceptional sensitivity to tumor acidity. PA signal amplification was retained across multiple imaging sessions, suggesting prolonged probe stability in vivo. SON‐based imaging confirmed precise tumor delineation with deep tissue penetration, establishing this system as a highly effective tool for real‐time pH‐responsive PAI and therapeutic monitoring.

Another notable strategy involves leveraging pH‐induced aggregation to enhance contrast in PAI. Gold nanoparticle‐based pH‐responsive probes developed by functionalizing c(RGDyk) peptides with citraconic amide‐based ligands (LSC) exhibit strong aggregation behavior under acidic conditions.^[132]^ These nanoparticles demonstrated a nearly threefold increase in PA signals in U87MG tumor models, with prolonged retention time and improved tumor imaging contrast. The dual‐ligand functionalization strategy enhances specificity by ensuring selective accumulation in tumor regions, reducing false positives, and increasing imaging accuracy. These findings highlight the effectiveness of pH‐induced aggregation in improving PAI contrast and selectivity.

To combine imaging and therapeutic applications, Chen et al. synthesized pH‐responsive Prussian blue hydrogels for combined PAI and PTT (Figure 8b).^[127]^ Synthesized by in situ hydrolysis in acidic environments, the hydrogels exhibited broad NIR absorption peaks at 720 nm, making them highly suitable for PA‐guided therapy. In vivo studies demonstrated strong PA signals, minimal toxicity, and effective tumor eradication under 808 nm irradiation. Under 808 nm laser irradiation, the temperature of the solution at 200 mM increased by 46°C in 10 min. The PA signal increased linearly with concentration, and NIR absorption decreased as the pH changed from 5.0 to 7.0, demonstrating the suitability of the agent for weak acidic conditions. Mice treated with PB‐based hydrogels exhibited extended survival with significantly reduced tumor volumes compared with untreated controls. The combination of PAI and PTT highlighted the versatility of PB‐based hydrogels for theranostic applications.

Seeking to increase the diagnostic accuracy of cancer biopsies, Choe et al. invented a PANI‐coated biopsy needle that indicated the pH level of the application site using ratiometric PAI.^[133]^ PANI exhibits pH‐sensitive spectral shifts between 700 and 850 nm, allowing real‐time tumor acidity quantification. The PA signal ratio (PA_850_/PA_700_) is a reliable metric for differentiating between malignant and healthy tissues. In vivo studies have demonstrated significant improvements in signal uniformity and biopsy accuracy, particularly for US‐guided procedures. This technology represents a promising advance in noninvasive tumor detection and precise biopsy guidance.

#### 3.2.3 Reactive oxygen/nitrogen species responsive

This section of the review surveys the design strategies and mechanisms of the ROS/RNS‐responsive probes, focusing on hydroxyl radicals (•OH), hydrogen peroxide (H_2_O_2_), superoxide anions (${O_{2^{•}}}^{-}$), and peroxynitrite (ONOO^−^). Although many of these probes target ROS owing to their diverse roles in oxidative stress and pathology, similar approaches have been applied to reactive nitrogen species (RNS), given their shared mechanistic pathways and relevance in biological systems.

**FIGURE 9:**
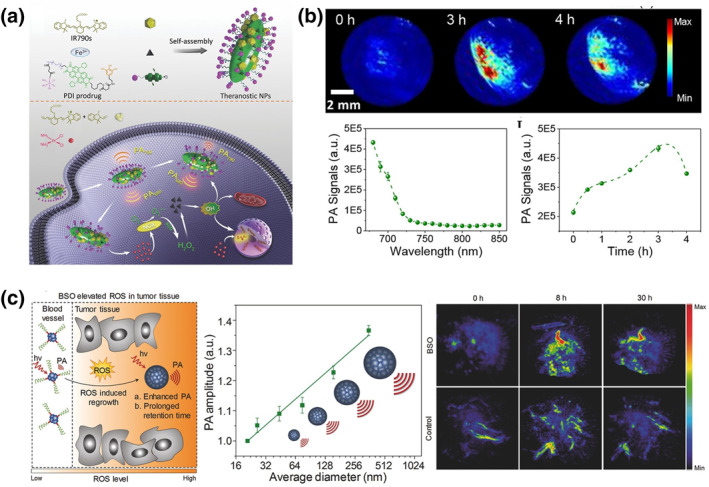
ROS‐responsive contrast agents. (a) PDI–IR790s–Fe/Pt NPs enabled activatable theranostic ROS imaging and therapy through pH‐responsive self‐assembly and Fenton reaction‐driven •OH production. Ratiometric PAI confirmed ROS generation in tumor‐bearing mice, demonstrating significant antitumor efficacy. Reproduced from ref. ^[134]^ with permission from John Wiley & Sons, copyright 2018. (b) CySO_3_CF_3_ functioned as a dual‐modal NIR fluorescence and PA probe for ONOO^−^ detection, activating upon oxidation with a red shift in absorption. Its high selectivity and minimal cytotoxicity enabled precise in vivo imaging and tumor localization. Reproduced from ref. ^[135]^ with permission from American Chemical Society, copyright 2018. (c) PCBP exhibited ROS‐responsive self‐assembly, enhancing PA signal intensity at 720 nm and enabling high‐sensitivity deep‐tissue imaging. Its structural transformation improved tumor retention, offering a promising platform for diagnostic imaging and therapy monitoring. Reproduced from ref. ^[136]^ with permission from American Chemical Society, copyright 2018. NIR, near‐infrared; PA, photoacoustic; PCBP, zinc‐tetra(2‐((1‐(3‐amino‐3‐oxopropyl)‐1H‐1,2,3‐triazol‐4‐yl)­methoxy)‐2‐oxo‐1‐(4‐((4‐(4,4,5,5‐tetramethyl‐1,3,2‐dioxaborolan‐2‐yl)benzyl)oxy)phenyl)ethyl methoxy poly(ethylene glycol) succinate)phthalocyanine; ROS, reactive oxygen species.

Among the diverse ROS‐responsive strategies, PDI–IR790s–Fe/Pt nanoparticles were designed as activatable theranostic platforms for ROS imaging and therapy (Figure 9a).^[134]^ These self‐assembled nanoparticles incorporate a perylene diimide (PDI)‐based cisplatin prodrug, IR790s dye, and chelated ferric ions that underwent ROS‐mediated activation. Exposed to tumor microenvironments, the nanoparticles release cisplatin and activate nicotinamide adenine dinucleotide phosphate oxidase to generate ${O_{2^{•}}}^{-}$ and H_2_O_2_, which is catalyzed by Fe^3+^ to produce toxic •OH via the Fenton reaction, significantly amplifying the PA signals. In vivo studies have demonstrated a robust PA signal increase in hypoxic tumor regions, effectively distinguishing them from normal tissues. This system exhibits strong therapeutic efficacy by combining ROS‐induced oxidative damage with cisplatin‐mediated apoptosis.

In a further extension of theranostic design, PcDA has been developed as a supramolecular phototherapeutic agent that integrates PDT and PTT with PAI.^[137]^ PcDa is constructed through electrostatic self‐assembly of cationic (PcD) and anionic phthalocyanines and undergoes Förster resonance energy transfer upon NIR irradiation, leading to enhanced ROS production and localized heating. PAI confirmed a strong signal contrast in the tumor regions that is correlated with effective photodynamic and photothermal therapeutic responses. Intravenous administration resulted in a PA signal contrast ratio of 11.9, enabling a clear distinction of the tumor region. The system demonstrated 95% tumor growth inhibition under combined PDT and PTT conditions, emphasizing its potential for multimodal cancer treatment.

Lipo@HRP and 2,2′‐azino‐bis(3‐ethylbenzothiazoline‐6‐sulfonic acid) (ABTS) liposomal nanoprobes were engineered for ROS‐responsive PAI and inflammation detection.^[138]^ These nanoprobes encapsulate horseradish peroxidase and ABTS, which undergo oxidation in the presence of H_2_O_2_, leading to a significant PA signal enhancement at 800 nm. The detection sensitivity reached as low as 0.8 μM, facilitating precise H_2_O_2_ quantification. In vivo imaging of inflammatory tissues revealed strong PA signal intensities, successfully distinguishing inflamed regions from normal tissues. Furthermore, the probe demonstrated promising photothermal therapeutic effects, achieving effective tumor suppression under NIR irradiation.

To address the need for dual‐mode imaging, CySO_3_CF_3_ was developed as an ONOO^−^ responsive probe for simultaneous PA and NIR FL imaging (Figure 9b).^[135]^ This hemicyanine‐based system undergoes a red‐shift absorption upon oxidation by ONOO^−^, enabling high‐contrast PA detection. The probe exhibited a rapid signal response and exceptional selectivity, achieving an 8‐fold increase in PA signal intensity in the ONOO^−^‐rich environments. In vivo studies have demonstrated clear tumor accumulation and enhanced contrast under inflammatory conditions, underscoring the utility of the probe in real‐time oxidative stress imaging. The probe successfully visualized ONOO^−^ levels in vivo, and the dual‐modal imaging enabled precise tumor localization with PAI, offering deep tissue penetration and enhanced FL spatial resolution.

Additionally, OEG‐Aza‐BODIPY‐BAPE was designed as a hydrophilic PA probe for the ratiometric imaging of H_2_O_2_ in biological systems.^[139]^ This probe integrates oligo(ethylene glycol) for enhanced solubility and an Aza‐BODIPY core decorated with an H_2_O_2_‐sensitive benzeneboronic acid pinacol ester (BAPE). Upon activation by H_2_O_2_, the probe exhibits dual PA absorption peaks at 700 and 850 nm, facilitating precise quantification of oxidative stress. In vitro, this probe showed high sensitivity toward H_2_O_2_, with minimal interference from other ROS and RNS species, achieving a 4.77‐fold enhancement in the ratiometric PA intensities. Strong probe accumulation was observed in ROS‐enriched tumor regions in vivo, further confirming the high sensitivity and specificity of PA‐based redox imaging.

Focusing on redox cycle monitoring, BDP‐DOH was designed as a reversible PA probe that dynamically responds to fluctuations in ${O_{2^{•}}}^{-}$ and GSH.^[140]^ This BODIPY‐derived probe undergoes oxidation upon exposure to ${O_{2^{•}}}^{-}$, shifting its PA absorption from 680 to 750 nm. The oxidation process is reversible upon GSH reduction, enabling continuous monitoring of the redox states. In vitro, BDP‐DOH showed an increase in the absorbance at 750 nm with increasing ${O_{2^{•}}}^{-}$ concentration, whereas increasing GSH concentration led to an increase at 680 nm and a decrease in the absorption at 750 nm. Comparison of the PA_750_/PA_680_ intensity with that of other analytes proved to be highly specific and sensitive. In tumor‐bearing mice, BDP‐DOH exhibited time‐dependent PA signal changes, allowing real‐time visualization of oxidative stress variations. The PA_750_/PA_680_ ratio of the tumor reached a maximum at 4 h after treatment and gradually decreased over time. This system demonstrated high biocompatibility and prolonged stability, making it a promising candidate for monitoring TME dynamics.

Another innovative approach was introduced using zinc‐tetra(2‐((1‐(3‐amino‐3‐oxopropyl)‐1H‐1,2,3‐triazol‐4‐yl)­methoxy)‐2‐oxo‐1‐(4‐((4‐(4,4,5,5‐tetramethyl‐1,3,2‐dioxaborolan‐2‐yl)benzyl)oxy)phenyl)ethyl methoxy poly(ethylene glycol) succinate)phthalocyanine (PCBP), which is a semiconducting macromolecular probe used for ROS imaging (Figure 9c).^[136]^ This phthalocyanine‐based system incorporates PEG linkers and ROS‐sensitive segments, leading to nanoparticle self‐assembly under high ROS conditions. ROS‐triggered structural transformations result in amplified PA signals, enabling high‐contrast deep tissue imaging. In vivo, PCB accumulates in tumors via the enhanced permeability and retention effect with a detection limit of 0.15 mM. Compared to the mice not treated with BSO (liposome preparation), BSO‐treated mice showed a 2.0‐fold higher PA signal, suggesting that increased ROS levels effectively activated the self‐assembly of PCBPs. Photoacoustic spectral profiles in the BSO‐treated mice were similar to those in the non‐treated mice; however, PA signals in the pretreated mice were 2.03 times higher, which was associated with the increased particle size and extended in vivo residence time.

Another dual‐modal imaging strategy was explored using ROS‐BODIPYs, which integrate PAI and FLI for oxidative stress monitoring.^[141]^ These conjugated BODIPY dyes undergo a reversible redshift in absorption from 700 to 800 nm upon oxidation, as well as a significant PA signal increase subsequent to FL quenching, enabling real‐time PA tracking of ROS fluctuations. Their reversible activation prevents probe accumulation, ensuring selective detection of pathological ROS levels. PAI demonstrations showed a 7‐fold PA signal enhancement upon activation, with a linear response in the 0.5–15 μM range of dye concentrations. The probe remained photostable under 5 min of continuous PA laser irradiation, confirming its suitability for long‐term monitoring. In A549 cells, ROS‐BODIPYs displayed a 6.8‐fold PA signal increase after menadione‐induced ROS production, demonstrating high selectivity. In vivo, activated probes exhibited a 4.9‐fold higher PA signal than the non‐activated probes, with spectral unmixing confirming precise ROS localization in the ROS‐enriched tissues.

Organic semiconducting nanoprobes (OSNs) were developed for ratiometric PAI of ONOO^−^ in tumors.^[142]^ The probes incorporate a boronate‐caged BODIPY dye that undergoes a red‐shift in absorption from 675 to 745 nm and a PA signal increase upon ONOO^−^ activation. To improve the selectivity, bulky borane dopants were added, preventing interference from H_2_O_2_ and ensuring ONOO^−^ specificity. PAI experiments showed an 8‐fold increase in ratiometric absorption (PA_745_/PA_675_) upon ONOO^−^ activation, whereas other ROS caused negligible changes. The sensitivity limit was measured as ∼100 nM, making OSNs highly sensitive for ONOO^−^ imaging. In vivo, tumor‐bearing mice showed a 2.6‐fold higher PA_750_/PA_680_ ratio in untreated tumors compared to the NAC‐pretreated (ROS‐scavenged) tumors, confirming selective ONOO^−^ detection. Spectral unmixing further validated precise ONOO^−^ localization in tumor regions.

#### 3.2.4 Glutathione (GSH) responsive

GSH is a key biomarker in the tumor microenvironment because of its elevated levels in cancer cells compared to normal tissues. This unique property has been widely utilized in the design of GSH‐responsive PAI probes, enabling precise tumor detection and diagnosis. Recent advances have addressed challenges such as low sensitivity, limited selectivity, and inadequate NIR absorption through innovative designs. Here, we summarize the notable studies on GSH‐activated PAI probes, focusing on the novel strategies, enhanced performance, and potential for precise and noninvasive tumor imaging.

Lucero et al. established a photoacoustic‐based companion diagnostic (PACDx) technology capable of measuring GSH levels in lung cancer models.^[143]^ It consists of a hemicyanine‐based dye that generates strong PA signals and a cleavage‐triggered GSH‐responsive moiety, with lipophilic cationic properties that enhance tumor cell uptake. Although the initial designs faced challenges in distinguishing GSH levels due to disulfide exchange and SNAr chemistry, the introduction of various electron‐withdrawing and electron‐donating groups improved probe sensitivity, achieving a dose‐dependent signal increase of up to 31.6‐fold. PACDx was validated in A549 xenograft lung cancer models, showing a 20% increase in the PA signal in tumor tissues compared to controls, demonstrating its high specificity and activation efficiency for GSH detection and imaging.

Wang et al. reported GSH‐responsive Fe‐Cu@PANI PAI probes that are activated through the redox reaction of Cu(II) with GSH in the tumor microenvironment.^[144]^ This process converts emeraldine based PANI to emeraldine‐salt PANI, generating strong NIR absorption and PA signals. Fe‐Cu@PANI nanoparticles were synthesized via an efficient and straightforward one‐pot method using BSA, FeCl_3_, and CuCl_2_, and exhibited a red‐shift in the absorption wavelength from 615 to 820 nm in the presence of GSH. In PAI experiments with 4T1 xenograft tumor models, Fe‐Cu@PANI showed a 20% stronger PA signal in tumor tissues than in normal tissues, with the signals persisting for 24 h post‐injection. These findings underscore the potential of Fe‐Cu@PANI as a diagnostic and therapeutic platform for targeting tumor GSH levels.

**FIGURE 10:**
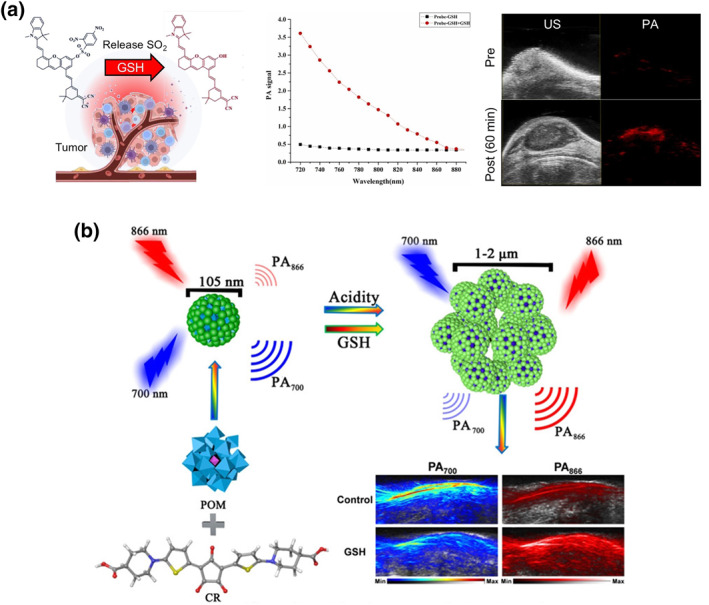
Glutathione (GSH)‐responsive contrast agents. (a) Probe‐GSH pairs a hemicyanine donor with a dicyanoisophorone acceptor and is unmasked by intracellular glutathione, yielding a broad 90 nm Stokes shift and a strong photoacoustic turn‐on at 720 nm. In 4T1 tumor‐bearing mice, this activation enables rapid, depth‐resolved 3D imaging that outperforms conventional NIR fluorescence for clear tumor delineation. Reproduced from ref. ^[145]^ with permission from Elsevier, copyright 2022. (b) CR‐POM self‐assembles croconaine dye with Mo‐based polyoxometalate clusters so that GSH concurrently quenches CR absorption at 700 nm while boosting POM absorption at 866 nm, producing a ratiometric PA_866_/PA_700_ read‐out. In U87MG tumors, this ratio rose to ∼1.3 within 8 h after injection, enabling selective, noninvasive quantification of intracellular GSH down to sub‐millimolar levels. Reproduced from ref. ^[146]^ with permission from American Chemical Society, copyright 2019. CR, croconaine; GSH, glutathione; NIR, near‐infrared; PA, photoacoustic; POM, polyoxometalate.

Qin et al. addressed the limitations of xanthene‐based hemicyanines, including low NIR absorption and small Stokes shifts, by developing a GSH‐triggered PA probe named Probe‐GSH (Figure 10a).^[145]^ The probe combines hemicyanine and dicyanoisophorone, offering a relatively large Stokes shift (Δ*λ* = 90 nm). The GSH‐responsive 2, 4‐dinitrobenzenesulfonyl chloride (DNS) moiety ensures selective activation. In the absence of GSH, the electron‐withdrawing effect of DNS suppressed PA signals, but activation by GSH generated up to an 8‐fold PA signal enhancement at 720 nm. In 4T1 xenograft mouse models, probe‐GSH achieved a rapid increase in the PA signal within 1 h post‐injection. Furthermore, PAI provided superior 3D tumor visualization compared to near‐infrared fluorescence (NIRF) imaging, offering more precise diagnostic information.

Tang et al. developed CR‐POM, an assembly of croconaine (CR) dye and Mo‐based polyoxometalate (POM) clusters, designed for ultrasensitive detection of intratumoral GSH via PA ratiometry (Figure 10b).^[146]^ These nanoparticles exhibited opposite absorbance shifts upon reaction with GSH. CR is reduced by GSH, decreasing its absorbance at 700 nm, whereas POM is reduced from Mo(VI) to Mo(V), increasing the absorbance at 866 nm. CR‐POM demonstrated high selectivity for GSH over other amino acids and achieved ratiometric PA signal changes (PA_866_/PA_700_) in U87MG xenograft tumor models, reaching a maximum value (1.33 ± 0.12) within 8 h post‐injection. Covering a GSH concentration range of 0.5–10 mM, CR‐POM exhibits a low limit of detection (LOD, 0.51 mM) and enables precise noninvasive GSH quantification.

Chen et al. introduced an in situ synthesized a nanozyme (ISSzyme) based on Prussian blue precursors that responds specifically to high GSH levels in the tumor microenvironments.^[147]^ ISSzyme converts into nanozyme of smaller aggregate size upon GSH reduction, which produces strong PA signals. ISSzyme induces spectral changes in response to GSH, resulting in up to 4.14‐fold linear increase in the absorbance ratios between 700 and 990 nm. In tumor models, ISSzyme enhanced the PA signal ratios (PA_700_/PA_990_) by a factor of 2.14, facilitating tumor‐specific imaging with high precision. Compared to the traditional Prussian blue nanozyme, ISSzyme exhibited significantly reduced liver accumulation, minimizing off‐target effects while demonstrating a 2.52‐fold improvement in the tumor‐to‐liver signal ratio.

#### 3.2.5 Enzyme responsive

Among the various PAI strategies, enzyme‐activated PA probes stand out for their ability to target tumor‐specific enzymes, maximizing the signal‐to‐background ratio while minimizing nonspecific signals. These probes are activated by enzymes to selectively generate strong PA signals in tumor regions, enabling precise and reliable imaging. This section highlights various enzyme‐activated PA probes targeting tumor‐related enzymes such as sulfatase, amino acids, hepsin, and caspase‐3, and discusses their design strategies and mechanisms.

**FIGURE 11:**
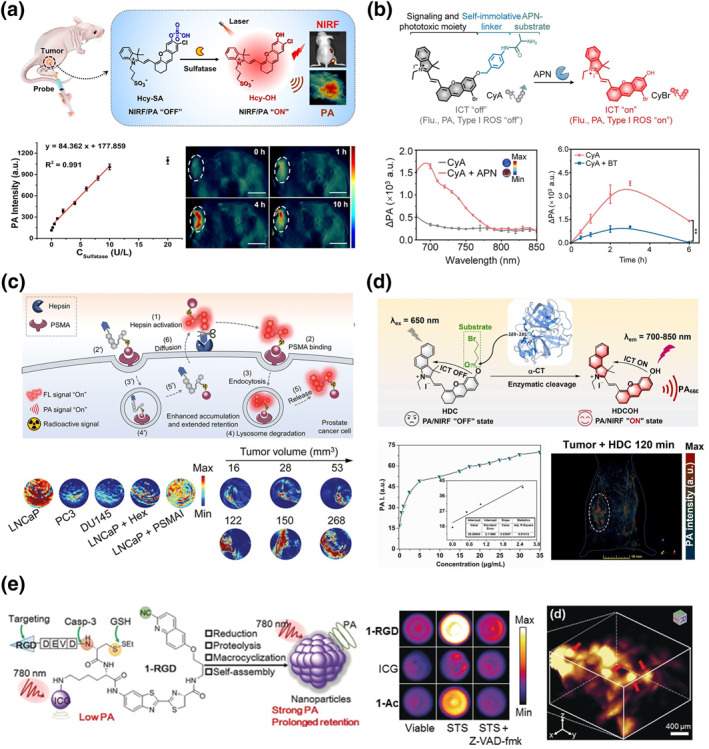
Enzyme‐responsive contrast agents. (a) Hcy‐SA is a sulfatase‐responsive PA/NIRF dual‐modal probe that activates via hydrolysis of its sulfate ester bond, restoring ICT and enhancing PA signals. Reproduced from ref. ^[148]^ with permission from American Chemical Society, copyright 2023. (b) CyA, an APN‐activated probe, generates PA and NIRF signals upon APN‐mediated cleavage of its alanine substrate, enabling specific detection of APN activity in hypoxic tumors. Reproduced from ref. ^[149]^ with permission from John Wiley & Sons, copyright 2023. (c) P‐^125^I is a hepsin‐ and PSMA‐responsive multimodal probe activated by hepsin cleavage, producing PA and NIRF signals while selectively targeting PSMA‐expressing prostate tumors. Reproduced with permission. Reproduced from ref. ^[150]^ with permission from John Wiley & Sons, copyright 2022. (d) HDC is an α‐chymotrypsin‐activated probe that restores ICT through selective cleavage, producing strong PA and NIRF signals for deep‐tumor imaging. Reproduced from ref. ^[151]^ with permission from Elsevier, copyright 2023. (e) 1‐RGD, a caspase‐3‐activated PA probe, undergoes macrocyclization and self‐assembly upon activation, amplifying PA signals for apoptosis detection. Reproduced from ref. ^[152]^ with permission from John Wiley & Sons, copyright 2019. APN, aminopeptidase N; HDC, Hemicyanine derivative for chymotrypsin; ICT, intramolecular charge transfer; NIRF, near‐infrared fluorescence; PA, photoacoustic; PSMA, prostate‐specific membrane antigen.

Hcy‐SA is an activatable NIRF/PA dual‐mode probe designed to detect sulfatase activity with high sensitivity (Figure 11a).^[148]^ Sulfatase is an enzyme that is commonly overexpressed in several cancers such as lung, liver, and breast cancer, which hydrolyzes sulfate ester bonds and plays a role in tumor progression. Hcy‐SA is comprised of a hemicyanine‐based NIRF/PA reporter and a sulfate ester moiety that can be cleaved by sulfatase. Sulfation of the –OH group inhibits the intramolecular charge transfer (ICT) effect. However, when cleaved by sulfatase, ICT is restored, activating the NIRF and PA signals. Hcy‐SA exhibited PA signal amplification proportional to sulfatase concentration, demonstrating high sensitivity and a low detection limit. In MCF‐7 tumor models, Hcy‐SA showed specific signal increases over time, validating its practical application in tumor imaging.

Zhao et al. developed CyA, a novel probe activated by aminopeptidase N (APN), an enzyme overexpressed in hypoxic tumor microenvironments (Figure 11b).^[149]^ CyA consists of a hemicyanine dye (CyBr) that is activated by the APN‐mediated cleavage of an alanine substrate, releasing CyBr and generating PA and NIRF signals. The PA signal intensity at 700 nm increased 4.9‐fold in the presence of APN. In 4T1 tumor‐bearing mice, PA signals progressively increased in the tumor regions over time, enabling precise spatial mapping of APN activity through PAI.

P‐^125^I is a multimodal probe specifically designed for the early diagnosis of prostate cancer. P‐^125^I integrates PA, NIRF, and radioactive imaging to target hepsin and prostate‐specific membrane antigen (PSMA; Figure 11c).^[150]^ P‐^125^I includes a hepsin‐cleavable peptide Ac‐KQLR, hemicyanine dye, and a PSMA‐targeting ligand labeled with radioactive iodine (^125^I). Upon cleavage by hepsin, the probe is activated to generate PA and NIRF signals, whereas the PSMA‐targeting feature enables selective accumulation in tumor tissues. In vitro, P‐^125^I generated strong PA signals in LNCaP cells that extensively expressed hepsin and PSMA. In vivo experiments showed that the signal intensity in the tumor regions increased proportionally with the tumor size in mouse models, effectively distinguishing prostate cancer with achieving a high renal clearance of 90.4%. P‐^125^I simultaneously targets two biomarkers, hepsin and PSMA, and integrates PA with NIRF and radioactive signals into a multimodal imaging system, achieving a significant improvement in diagnostic accuracy and applicability.

Hemicyanine derivative for chymotrypsin (HDC) is a non‐peptide‐based α‐chymotrypsin (α‐CT) activated probe developed for precise diagnosis with 3D PAI (Figure 11d).^[151]^ The probe consists of a hemicyanine dye and a 4‐bromobutyryl acetyl group, and is selectively cleaved by α‐CT to restore the ICT mechanism. This activation triggers the hemicyanine dye to emit strong PA and NIRF signals in the 700–900 nm range. HDC exhibits a linear correlation between PA/NIRF signal intensity and α‐CT concentration, achieving a high sensitivity with a detection limit of 72 ng/mL. In P815 tumor‐bearing mice, HDC demonstrated increased PA and NIRF signals in tumor regions, effectively visualizing tumor depth and spatial distribution using 3D PAI. Compared to peptide‐based probes, the non‐peptide design of HDC enhances in vivo stability and facilitates accurate diagnosis of deep‐seated tumors by specifically targeting α‐CT.

Wang et al. developed an activatable PA probe nanoparticle, 1‐RGD, which was designed to detect caspase‐3 activity and visualize tumor cell apoptosis (Figure 11e).^[152]^ This novel tool enables noninvasive real‐time monitoring of chemotherapy‐induced tumor cell death, providing an early evaluation of therapeutic efficacy. 1‐RGD consists of a caspase‐3‐cleavable peptide, c‐RGD ligand for tumor cell targeting, and ICG. Upon caspase‐3 activation, 1‐RGD undergoes macrocyclization and self‐assembly into nanoparticles. These nanoparticles suppress the FL signals through aggregation‐caused quenching while amplifying the PA signals. Caspase‐3 activation results in an approximately 3‐fold increase in the PA signal intensity, and the transformation into nanoparticles prolongs the retention of the probe in tumor tissue, enhancing signals at apoptosis sites, achieving high specificity and efficiency for caspase‐3 detection. In the DOX‐induced U87MG tumor model, 1‐RGD achieved up to 4.4‐fold PA signal amplification in apoptotic regions, with 3D PAI enabling detailed visualization of apoptosis within tumors.

Table 2 lists a comprehensive overview of the methodologies and key findings of the selected studies.

**TABLE 2:** Summary of microenvironment‐triggered photoacoustic contrast agents.

Ref	Stimuli	Name	Condition	Switching mechanism	Multimodality	Peak absorption wavelength (nm)	Molar extinction coefficient (on/off) (×10^4^ M^−1 ^cm^−1^)	Imaging wavelength (nm)	PA signal switching ratio	Target application
^[122]^	Hypoxia	HyP‐1	O_2_ absence by heme proteins	Enzymatic reduction of 2‐nitroimadazoles	PA, FL	760	5.4	770	∼20.5	Tumor imaging
^[123]^	Hypoxia	TBTO	Reductase	Twisted intramolecular charge transfer	PA, FL	542	1.8	900	‐	Tumor imaging
^[124]^	Hypoxia	NOx‐JS013	O_2_ absence by heme proteins	Hypoxia specific enzyme; tumor specific enzyme binding	PA	668	9.63	770	14.1	Tumor imaging
^[125]^	Hypoxia	IR1048‐MZ	Nitroreductase (NTR)	Reduction of IR1048‐MZ by NTR	PA, NIR FL	980	‐	880	∼10	Tumor imaging and treatment
^[126]^	pH	SNARF‐5	pH 5.8–7.8	Dual wavelength ratiometric shift	PA	600	‐	565–600	2.5–4.0	Tumor imaging
^[128]^	pH	PANI‐BSA	pH 5–6.8	Polyaniline state alteration	PA	800 (acidic), 570 (basic)	‐	800	∼1.8	Tumor imaging
^[129]^	pH	CrocRot	pH 5.0–6.5	Croconaine redshift	PA	794 (acidic), 660 (basic)	40	740	∼5	Tumor imaging
^[130]^	pH	HSA‐BPOx‐IR825	pH 5.0–7.0	BPOx redshift	PA	680	‐	680/825	∼2.5	Tumor imaging
^[131]^	pH	SON	pH 5.8–7.4	pH‐BDP absorption decrease with protonation	PA	680 (acidic), 750 (basic)	‐	680/750	∼3.1	Tumor monitoring
^[132]^	pH	c(RGDyk)‐MHDA/LSC@AuNP	pH 5.8–7.4	Triggered aggregation with citraconic amide hydrolysis	PA	680	‐	680	∼3.6	Tumor monitoring
^[127]^	pH	Prussian blue hydrogel	pH 5.0–6.0	Prussian blue release from acidic hydrogel	PA, PTT	720	‐	720	∼3.0	Tumor monitoring
^[133]^	pH	PANI‐needle	pH 6.5–7.5	PANI redshift	PA	850 (acidic), 700 (basic)	‐	850/700	∼1.3	Tumor biopsy guidance
^[134]^	ROS/RNS	PDI–IR790s–Fe/Pt NPs	•OH	Fenton reaction activates IR790	PA	680, 790	‐	680/790	∼4	Tumor monitoring
^[137]^	ROS/RNS	PcDA	ROS	FRET enhancing ROS generation and heat	PAI, PTI	655, 690	5.8	655, 690	∼11.9	Tumor imaging and treatment
^[138]^	ROS/RNS	Lipo@HRP&ABTS	H_2_O_2_	HRP uses H_2_O_2_ to oxidize ABTS	PA	800	‐	PA	∼20	Inflammation imaging and tumor treatment
^[135]^	ROS/RNS	CySO_3_CF_3_	ONOO^−^	Trifluoromethyl ketone oxidation	PA, NIR FL	686	‐	680	∼5.1	Tumor imaging
^[139]^	ROS/RNS	Aza‐BODIPY‐BAPE	H_2_O_2_	H_2_O_2_ oxidizes benzeneboronic acid	PA	720, 825	1.2 (720 nm)	825/720	∼4.77	Tumor imaging
^[140]^	ROS/RNS	BDP‐DOH	${O_{2^{•}}}^{-}$, GSH (reversible switching)	BDP‐DOH oxidation/reduction by ROS and GSH	PA	680 (reduced), 750 (oxidized)	4.2 (680 nm)	750/680	∼9.84	Tumor monitoring
^[136]^	ROS/RNS	PCBP	ONOO^−^, H_2_O_2_	PEG cleavage	PA	700	‐	700	∼1.4	Tumor monitoring
^[141]^	ROS/RNS	ROS‐BODIPY	ROS	BODIPY absorption shift	PA	700 (inactive), 810 (active)	6.9 (inactive), 8.0 (active)	875	∼7	Tumor imaging
^[142]^	ROS/RNS	OSN	ONOO^−^	ONOO^−^ cleaves boronate ester	PA	675 (inactive), 745 (active)	‐	750/680	∼7	Tumor monitoring
^[143]^	GSH	PACDx	GSH	GSH‐responsive cleavage of a sulfonyl trigger	PA	690	‐	680/900	1.2	Tumor imaging and treatment
^[144]^	GSH	Fe‐Cu@PANI	GSH	The redox reaction between Cu(II) and GSH	PA	∼820	‐	820	∼2	Tumor imaging and treatment
^[145]^	GSH	Probe‐GSH	GSH	Intramolecular charge transfer process by DNS moiety cleavage	PA	720	‐	720	8	Tumor imaging
^[146]^	GSH	CR‐POM	GSH	Reduction of Mo‐based POM by GSH	PA, PET	866	‐	866/700	‐	Tumor monitoring
^[147]^	GSH	ISSzyme	GSH	Synthesis of PB nanozyme by GSH	PA	∼700	‐	700/990	2.17	Tumor imaging and treatment
^[148]^	Enzyme	Hcy‐SA	Sulfatase	Intramolecular charge transfer through sulfatase‐catalyzed hydrolysis	PA, NIRF	685	‐	680	5.5	Tumor imaging
^[149]^	Enzyme	CyA	Aminopeptidase N	Conversion of CyA to CyBr after APN hydrolysis	PA, NIRF	692	3	700	3.2 decrease	Tumor imaging and treatment
^[150]^	Enzyme	P‐^125^I	Hepsin	Recovery of signaling moiety via hepsin‐cleavable substrate release	PA, NIRF	695	‐	700	‐	Tumor monitoring
^[151]^	Enzyme	HDC	α‐CT	α‐CT cleaves ester, restoring ICT	PA, NIRF	703	‐	660	∼7	Tumor monitoring
^[152]^	Enzyme	1‐RGD	Caspase‐3	ICG self‐assembly induced by caspase‐3 and GSH‐triggered cyclization	PA	780	‐	780	22	Tumor monitoring

Abbreviations: BPOx, benzo‐a‐phenoxazine; DNS, 2, 4‐dinitrobenzenesulfonyl chloride; FL, fluorescence; FRET, Förster resonance energy transfer; GSH, glutathione; HRP, horseradish peroxidase; ICT, intramolecular charge transfer; NIRF, near‐infrared fluorescence; PA, photoacoustic; PANI, polyaniline; PCBP, zinc‐tetra(2‐((1‐(3‐amino‐3‐oxopropyl)‐1H‐1,2,3‐triazol‐4‐yl)­methoxy)‐2‐oxo‐1‐(4‐((4‐(4,4,5,5‐tetramethyl‐1,3,2‐dioxaborolan‐2‐yl)benzyl)oxy)phenyl)ethyl methoxy poly(ethylene glycol) succinate)phthalocyanine; PDT, Photodynamic therapy; PEG, polyethylene glycol; POM, polyoxometalate.

## 4 CONCLUSION AND OUTLOOK

Our review highlights trigger‐responsive PAI contrast agents that exhibit active, switchable features responding to external stimuli or microenvironmental cues. By leveraging the unmatched penetration depth of PAI, complementary external triggers can reach several millimeters to centimeters, enabling non‐invasive real‐time monitoring of probe activation alongside functionalities such as multimodal integration, remote actuation, and synergistic theranostics. Activatable probes further extend the functional scope of PAI by sensitively reporting physiological changes and offering access to new classes of biomarkers beyond chromophore density.

Compared with conventional clinical contrast agents, activatable PAI probes offer a distinctive balance of depth and molecular specificity. Conventional optical probes, such as FL dyes, are already indispensable in confocal microscopy and FL‐guided surgery, providing nanomolar sensitivity and cellular resolution for monitoring ion kinetics, protease activity, and pH. However, their penetration is fundamentally limited to superficial tissues, restricting their applicability for deep‐seated lesions. At the other end of the spectrum, CT and MR angiographic contrast agents deliver robust whole‐body anatomical coverage and high spatial resolution, but they are largely nonspecific, report mainly perfusion or vascular morphology, and often require high systemic doses associated with safety concerns such as nephrotoxicity or gadolinium retention. Activatable PAI probes bridge these two domains. They extend the molecular sensitivity of optical methods to depths of several millimeters to centimeters, enabling longitudinal imaging of disease processes without repeated surgical sampling. At the same time, they can be engineered to respond to biochemical cues—such as enzyme activity or redox imbalance—providing molecular information that radiological agents cannot offer. Despite challenges, including background absorption from hemoglobin and melanin, resolution limits imposed by acoustic diffraction, and regulatory unfamiliarity, the ability of PAI to uniquely combine depth, molecular specificity, and noninvasiveness positions it as an irreplaceable complement within the current diagnostic landscape.

The next frontier for activatable PA contrast agents extends into the realm of clinically tailored probes that can reveal previously inaccessible biomarkers with meaningful depth, molecular specificity, and diagnostic reliability. Moving beyond proof‐of‐concept imaging in superficial tumor xenografts, future efforts should prioritize disease‐tailored probe design aimed at enabling noninvasive diagnosis of clinically demanding pathologies that currently necessitate surgical access. A particularly instructive example can be found in atherosclerosis, where activatable probes have already demonstrated the ability to delineate lipid‐ and collagen‐rich plaques, detect oxidative stress, and map enzyme‐driven inflammatory activity at centimeter depths, providing an early, noninvasive indication of cardiovascular risk prior to surgical intervention.[^153^, ^154^, ^155^, ^156^, ^157^, ^158^] Building on this paradigm, similar strategies could be applied to other diagnostically challenging pathologies where lesion accessibility is limited, such as sentinel lymph node metastasis, bowel wall inflammation, and neurovascular diseases. By capitalizing on the superior penetration of PAI, such approaches could reduce the need for repeated surgical inspections for diagnostic confirmation, enable longitudinal monitoring to assess recovery after intervention, and potentially extend toward theranostic applications by coupling molecular imaging with targeted drug delivery.

To translate these applications into practice, several key challenges must be addressed. First, probes must achieve biocompatibility, predictable pharmacokinetics, and sufficient light stability as many current agents based on synthetic dyes or nanocarriers show uncertain clearance profiles and limited photostability. Carriers that penetrate diseased tissue yet clear rapidly through renal or hepatobiliary routes will be essential to minimize long‐term toxicity. Second, the lack of standardization in probe characterization, imaging protocols, and quantitative metrics hampers clinical translation, underscoring the need for interdisciplinary cooperation among chemists, engineers, clinicians, and regulators. In parallel, advances in PAI hardware are equally important. As PAI remains a niche modality, future improvements in areas such as real‐time fluence correction and acoustic deaberration will be needed to enhance the accuracy and clinical reliability of video‐rate quantitative PAI. Furthermore, hybrid constructs that integrate PA contrast with MRI or PET reporters could further enhance sensitivity through multimodal imaging and facilitate regulatory approval.

## CONFLICT OF INTEREST STATEMENT

C.K. has financial interests in OPTICHO, which, however, did not support this work. The other authors declare no conflicts of interests.
